# Different Gabapentin and Pregabalin Dosages for Perioperative Pain Control in Patients Undergoing Spine Surgery: A Systematic Review and Network Meta-Analysis

**DOI:** 10.1001/jamanetworkopen.2023.28121

**Published:** 2023-08-09

**Authors:** Sung Huang Laurent Tsai, Ching-Wei Hu, Sally El Sammak, Sulaman Durrani, Abdul Karim Ghaith, Che Chung Justin Lin, Ewa Zuzanna Krzyż, Mohamad Bydon, Tsai Sheng Fu, Tung Yi Lin

**Affiliations:** 1 Department of Orthopaedic Surgery, Chang Gung Memorial Hospital, Keelung Branch, Taiwan; 2 School of Medicine, Chang Gung University, Taoyuan, Taiwan; 3 Mayo Clinic Neuro-Informatics Laboratory, Mayo Clinic, Rochester, Minnesota; 4 Department of Neurological Surgery, Mayo Clinic, Rochester, Minnesota; 5 School of Nursing, National Taipei University of Nursing and Health Sciences, Taipei, Taiwan; 6 Department of Orthopaedic Surgery, Chang Gung Memorial Hospital, Linkou Branch, Taiwan

## Abstract

**IMPORTANCE:**

Patients undergoing spine surgery often experience severe pain. The optimal dosage of pregabalin and gabapentin for pain control and safety in these patients has not been well established.

**OBJECTIVE:**

To evaluate the associations of pain, opioid consumption, and adverse events with different dosages of pregabalin and gabapentin in patients undergoing spine surgery.

**DATA SOURCES:**

PubMed/MEDLINE, Embase, Web of Science, Cochrane library, and Scopus databases were searched for articles until August 7, 2021.

**STUDY SELECTION:**

Randomized clinical trials conducted among patients who received pregabalin or gabapentin while undergoing spine surgery were included.

**DATA EXTRACTION AND SYNTHESIS:**

Two investigators independently performed data extraction following the PRISMA (Preferred Reporting Items for Systematic Reviews and Meta-analyses) reporting guideline. The network meta-analysis was conducted from August 2022 to February 2023 using a random-effects model.

**MAIN OUTCOMES AND MEASURES:**

The primary outcome was pain intensity measured using the Visual Analog Scale (VAS), and secondary outcomes included opioid consumption and adverse events.

**RESULTS:**

Twenty-seven randomized clinical trials with 1861 patients (median age, 45.99 years [range, 20.00-70.00 years]; 759 women [40.8%]) were included in the systematic review and network meta-analysis. Compared with placebo, the VAS pain score was lowest with gabapentin 900 mg per day, followed by gabapentin 1200 mg per day, gabapentin 600 mg per day, gabapentin 300 mg per day, pregabalin 300 mg per day, pregabalin 150 mg per day, and pregabalin 75 mg per day. Additionally, gabapentin 900 mg per day was found to be associated with the lowest opioid consumption among all dosages of gabapentin and pregabalin, with a mean difference of −22.07% (95% CI, −33.22% to −10.92%) for the surface under the cumulative ranking curve compared with placebo. There was no statistically significant difference in adverse events (nausea, vomiting, and dizziness) among all treatments. No substantial inconsistency between direct and indirect evidence was detected for all outcomes.

**CONCLUSIONS AND RELEVANCE:**

These findings suggest that gabapentin 900 mg per day before spine surgery is associated with the lowest VAS pain score among all dosages. In addition, no differences in adverse events were noted among all treatments.

## Key Points

**Question:**

What is the association of different dosages of pregabalin and gabapentinoids with pain control and adverse outcomes in patients undergoing spine surgery?

**Findings:**

In this systematic review and network meta-analysis of 27 randomized clinical trials with 1861 patients, gabapentin 900 mg per day was associated with the lowest Visual Analog Scale pain score and was found to be the best in terms of reducing opioid consumption, followed by gabapentin 1200 mg per day, gabapentin 600 mg per day, gabapentin 300 mg per day, pregabalin 300 mg per day, pregabalin 150 mg per day, and pregabalin 75 mg per day. No statistically significant difference in adverse events (ie, nausea, vomiting, and dizziness) was noted among all treatments.

**Meaning:**

These findings may guide clinicians and surgeons in determining the appropriate dosage of pregabalin and gabapentinoids in patients undergoing spine surgery.

## Teaser

This systematic review and network meta-analysis evaluates the associations of pain, opioid consumption, and adverse events with different dosages of pregabalin and gabapentin in patients undergoing spine surgery.

## Introduction

Successful pain management for patients undergoing spine surgery is essential for patient satisfaction. Adequate postoperative pain control has been linked to better outcomes, lower opioid consumption, shorter hospital stays, and reduced costs.^1,2^ Multimodal analgesia is currently the standard of care for postoperative pain management, and gabapentinoids are often used as part of this approach to decrease neuropathic pain.^3,4^

Gabapentinoids, such as gabapentin and pregabalin, can inhibit central nervous sensitization. Although these 2 drugs have similar mechanisms of action and chemical structures, pregabalin is more potent and takes effect more quickly than gabapentin.^5^ In recent years, high-quality evidence has demonstrated the effectiveness and safety of using gabapentinoids to treat neuropathic pain following spinal cord injury.^6,7^ A previous meta-analysis^8^ with limited sample size and no head-to-head comparisons found that both gabapentin and pregabalin were effective in reducing postoperative pain and opioid consumption following spine surgery compared with placebo. However, new trials with direct comparisons have been published, and the results are conflicting. The aim of this study is to conduct a systematic review and meta-analysis to compare the effectiveness and safety of gabapentin and pregabalin for spine surgery in the perioperative period.

## Methods

### Research Protocol and Search Question

We followed the PICO (problem, intervention, comparison, outcome) search protocol framework to investigate the use of gabapentin and pregabalin in patients undergoing spinal surgery. The primary outcome of our study was pain intensity measured using the Visual Analog Scale (VAS), with secondary outcomes including adverse events (ie, nausea, vomiting, and dizziness) and opioid consumption. We adhered to Preferred Reporting Items for Systematic Reviews and Meta-analyses (PRISMA) reporting guideline and registered the study in PROSPERO (CRD42020188512). This study is a systematic review and meta-analysis conducted in accordance with the guidelines outlined by the Common Rule, which exempts this research from the requirement of institutional review board approval.

### Eligibility Criteria

We included studies involving adult patients undergoing spine surgery with specific doses of gabapentin or pregabalin. We excluded single-group studies, case reports, basic science experiments, and animal or cadaver studies, as well as studies with patients with severe infection or under immunosuppression. We also excluded conference abstracts without full-length articles.

### Search Strategy and Study Selection

The electronic database search strategy is outlined in eTable 1 in Supplement 1. We conducted a comprehensive search using controlled vocabulary and keywords in Ovid/MEDLINE, Embase, Cochrane CENTRAL, Cochrane database of systematic reviews, and Scopus for randomized clinical trials (RCTs), up to August 2021. Two reviewers (S.H.L.T., C.W.H.) independently screened titles, abstracts, and full-text articles. Disagreements were resolved through discussion and consultation with a third reviewer (T.Y.L.) when necessary.

### Data Collection and Quality Assessment

Two independent reviewers (S.H.L.T. and C.W.H.) extracted data onto a preplanned Excel (version 2013; Microsoft) spreadsheet, including study characteristics, patient demographics, outcomes, and funding sources. Pain intensity was standardized using VAS scores ranging from 0 to 10 (with higher scores indicating worse pain), and opioid consumption was measured in milligrams and all data were converted into morphine milligram equivalents. We considered the concept of minimum clinically important differences (MCIDs) and assessed the quality of included studies using the Cochrane risk-of-bias tool.^9,10,11^ We conducted a literature review to determine the MCID values for pregabalin and gabapentin and indicated an MCID of 1.5 points on the VAS for these medications.^12,13^ The IQR was divided by 1.35 as approximate SD data when SD was not available.^14^

### Statistical Analysis

We performed network meta-analysis using Stata statistical software version 17 (StataCorp) to estimate treatment effects. Forest plots were used to compare treatments with placebo as the reference group. Heterogeneity was assessed using τ^2^ and *I*^2^ values. We used the surface under the cumulative ranking curve (SUCRA) to assess treatment performance.^15^ The SUCRA value closer to 100% indicates a higher probability of a treatment being among the top-ranked treatments or the best option overall.^16^ Inconsistency between direct and indirect comparisons was evaluated using the design-by-treatment interaction model.^17^ Also, Egger test and a funnel plot were performed for small-study bias.^18,19,20^ In our study, we used a meta-regression analysis to explore associations between study-level characteristics and treatment effects. Meta-regression analysis is a statistical method commonly used to investigate the associations of various factors with treatment effects across multiple studies. By considering study-level characteristics as covariates, we aimed to explore how these factors may be associated with treatment effects.^14,21,22^ A network meta-regression analysis extends these concepts by incorporating both treatment comparisons and covariates simultaneously. It allows for the examination of how treatment effects may be influenced by various factors, such as patient characteristics, study characteristics, or treatment characteristics. By considering these effect modifiers, network meta-regression can provide additional insights into the factors that may impact treatment effects and help explain the heterogeneity observed across studies. Levels of evidence are defined according to Halperin et al.^23^ Last, we used a semiautomated web application, Confidence in Network Meta-analysis (CINeMA),^24^ for the certainty of evidence for each outcome.

## Results

### Literature Search and Selection Process

**Figure 1.**
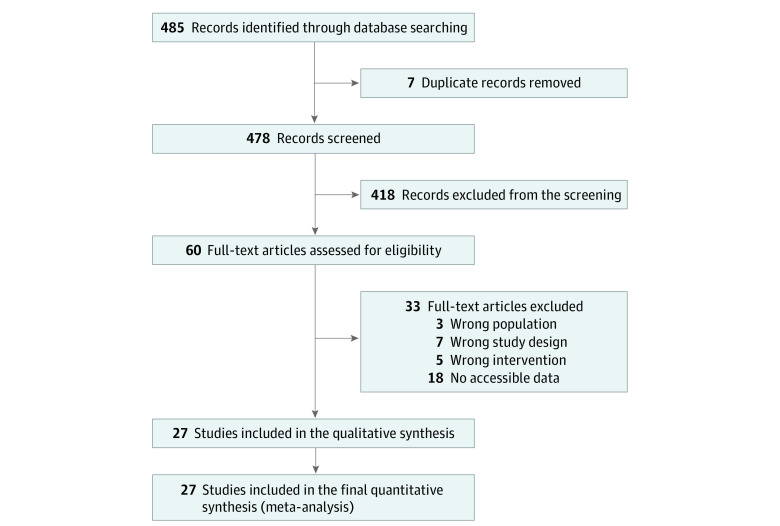
Preferred Reporting Items for Systematic Reviews and Meta-Analyses Flow Diagram Illustrating the Study Selection Process The flow diagram shows the number of studies identified through the initial literature search, the number of studies screened for eligibility based on predetermined criteria, the number of studies included in the qualitative synthesis, and the number of studies included in the quantitative analysis. The diagram helps to track the selection process and provides transparency in the study selection procedure.

A total of 485 articles were identified through the database search. After the removal of duplicates, 478 articles remained. Next, 418 articles were excluded by checking the titles and abstracts. After checking the full text of the remaining studies, 33 articles were excluded according to exclusion criteria and mismatching of inclusion criteria, with details listed in eTable 2 in Supplement 1. Ultimately, 27 studies were included in the network meta-analysis as shown in the flowchart (Figure 1).

### Study Characteristics and Description

**Figure 2.**
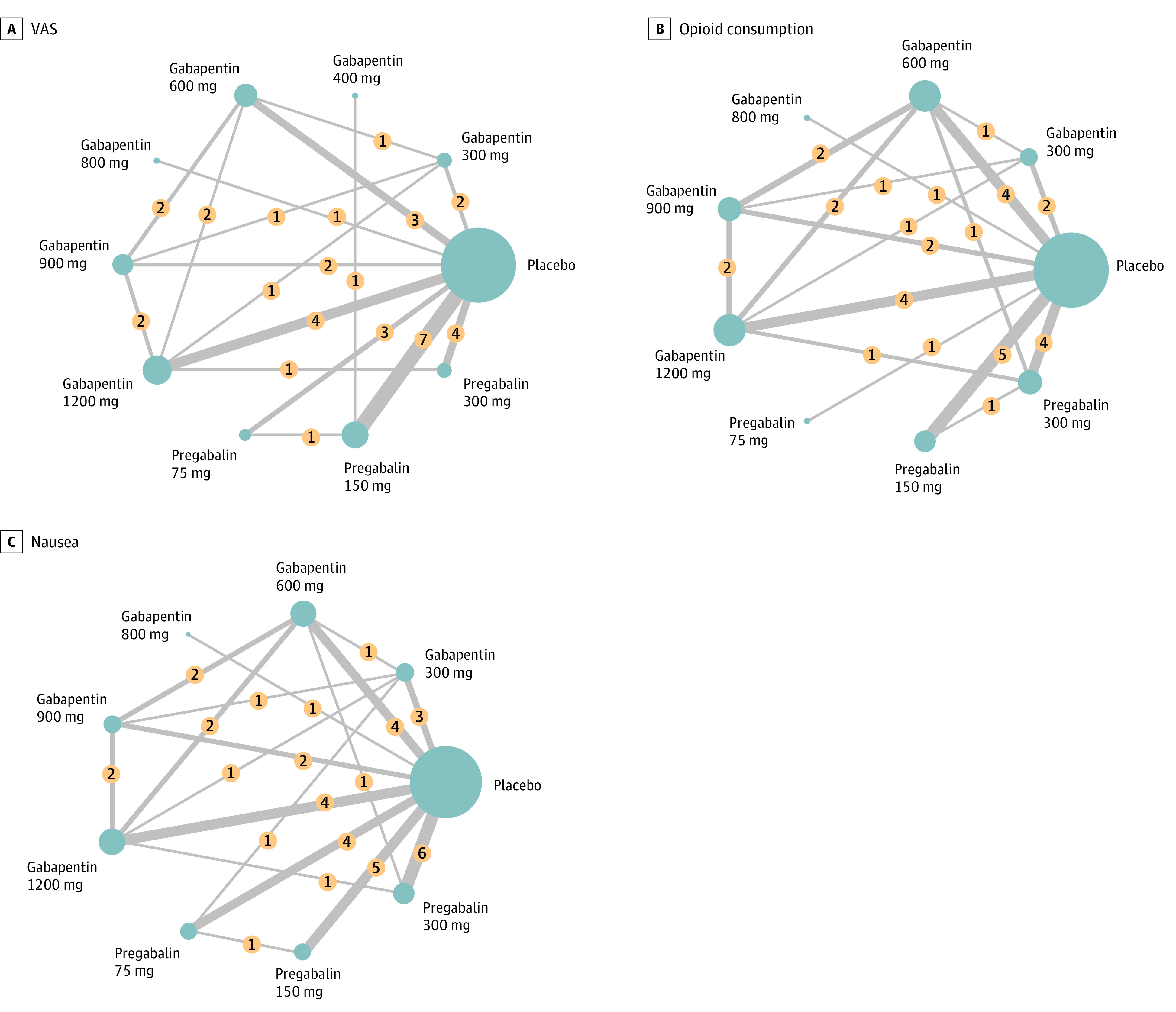
Network Structure Diagram Illustrating the Connections Between Interventions Based on Direct and Indirect Evidence Obtained From the Included Studies Each node in the diagram represents a specific intervention, whereas the lines connecting the nodes depict the available comparisons between interventions. The network structure provides a visual representation of the relationships and comparisons among the interventions. In this figure, we present the network structure for 3 specific outcome measures: postoperative Visual Analog Scale (VAS) score for pain intensity (A), postoperative opioid consumption (B), and postoperative nausea (C). Numbers in yellow circles denote the study numbers for the comparison of interventions. The thickness of the lines indicates the study numbers for the comparison of interventions. The sizes of the blue circles indicate the total patient number for each intervention.

Our network meta-analysis included 27 RCTs with a total of 1861 patients (median age, 45.99 years [range, 20.00-70.00 years]; 802 women [43.1%]) including 777 patients who used placebo, included in the analysis.^25,26,27,28,29,30,31,32,33,34,35,36,37,38,39,40,41,42,43,44,45,46,47,48,49,50,51^ The network graphs are presented in Figure 2 and eFigure 1 in Supplement 1, and the main characteristics of the included studies are reported in Table 1. The included studies were conducted in Asia (21 studies; 1528 patients),^25,27,28,32,36,37,38,39,40,41,42,45,46,47,48,49,50^ the US (1 study; 86 patients),^43^ and Europe (5 studies; 247 patients).^29,30,33,35,47^ eTable 3 in Supplement 1 provides detailed information regarding the use of gabapentinoids and nonsteroidal anti-inflammatory drugs before the study, preexisting neuropathic pain, the duration of perioperative administration of gabapentinoids, the timing of outcome assessments, reported comorbidities, comedications, and other outcomes. The transitivity was acceptable owing to insignificant variability identified in the study, population baselines, and network graph structure. Detailed risk of bias assessments is presented in eFigure 2 in Supplement 1. Some studies^29,30,40,43,46,50^ had funding from pharmaceutical companies, research centers from universities, and relevant departments from hospitals and foundations (Table 1).

**Table 1.** Characteristics of Included Studies^a^

Source	Country	Study type, level of evidence	Funding	Surgery	Preemptive, intraoperative, and postoperative treatment, mg/d per os	Postoperative parenteral opioid consumption	Total patient No.	Age, mean (SD) or mean (range), y
Pandey et al,^26^ 2004	India	RCT, I	No	Lumbar discectomy	Placebo	Fentanyl	56	39.1 (11.6)
					Gabapentin 300, NA, NA			38.5 (7.7)
Turan et al,^27^ 2004	Turkey	RCT, I	No	Lumbar discectomy or spinal fusion	Placebo	Morphine	50	45 (8)
					Gabapentin 1200, NA, NA			48 (9)
Pandey et al,^28^ 2005	India	RCT, I	No	Lumbar discectomy	Placebo	Fentanyl	100	39.6 (11.5)
					Gabapentin 300, NA, NA			39.9 (11.1)
					Gabapentin 600, NA, NA			40.7 (12)
					Gabapentin 900, NA, NA			43.5 (11.1)
					Gabapentin 1200, NA, NA			42.3 (12.8)
Radhakrishnan et al,^29^ 2005	India	RCT, I	No	Lumbar discectomy and laminectomy	Placebo	Morphine	60	41.67 (12.06)
					Gabapentin 800, NA, NA			39.63 (10.87)
Burke et al,^30^ 2010	Ireland	RCT, I	Yes	Lumbar discectomy	Placebo	Morphine	38	41 (12.4)
					Pregabalin 300, NA, 150			37 (7.8)
Hegarty et al,^31^ 2011	Ireland	RCT, I	Yes	Lumbar discectomy	Placebo	Morphine	32	41 (8.1)
					Pregabalin 300, NA, NA			38.8 (7.9)
Khan et al,^32^ 2011	Iran	RCT, I	No	Lumbar laminectomy	Placebo	Morphine	175	41.0 (10.5)
					Gabapentin 600, NA, NA			43.6 (10.8)
					Gabapentin NA, NA, 600^b^			43.5 (8.7)
					Gabapentin 900, NA, NA			41.9 (10.7)
					Gabapentin NA, NA, 900^b^			41.1 (10.2)
					Gabapentin 1200, NA, NA			40.4 (10.3)
					Gabapentin NA, NA, 1200^b^			41.0 (10.2)
Kim et al,^33^ 2011	South Korea	RCT, I	No	Lumbar spinal fusion	Placebo	Morphine	84	38 (33-48)^c^
					Pregabalin 75, NA, NA			
					Pregabalin 150, NA, NA			
Spreng et al,^34^ 2011	Norway	RCT, I	No	Lumbar discectomy	Placebo	Morphine	46	42.9 (7.6)
					Pregabalin 150, NA, NA			44.1 (10.8)
Ozgencil et al,^35^ 2011	Turkey	RCT, I	No	Lumbar discectomy and laminectomy	Placebo	Morphine	90	48.6 (6.5)
					Gabapentin 1200, NA, 1200			50.6 (9.1)
					Pregabalin 300, NA, 300			51.9 (7.1)
Gianesello et al,^36^ 2012	Italy	RCT, I	No	Lumbar laminectomy and spinal fusion	Placebo	Morphine	60	66.2 (10.8)
					Pregabalin 300, NA, 300			63.5 (9.9)
Choi et al,^37^ 2013	South Korea	RCT, I	No	Lumbar laminectomy or spinal fusion	Placebo	Fentanyl	108	54 (21-69)
					Pregabalin 300, NA, NA			53 (25-70)
					Pregabalin 300, NA, NA plus dexamethasone 16 mg, NA, NA^b^			52 (20-69)
Kumar et al,^38^ 2013	India	RCT, I	No	Lumbar laminectomy	Placebo	Fentanyl	75	45.64 (11.1)
					Pregabalin 150, NA, NA			45.36 (11.04)
					Tramadol 100, NA, NA^b^			41.8 (12.43)
Khurana et al,^39^ 2014	India	RCT, I	No	Lumbar discectomy	Placebo	NA	90	47.1 (10.7)
					Gabapentin 300, NA, 900			49 (10.4)
					Pregabalin 75, NA, 225			46.9 (10.1)
Zarei et al,^40^ 2016	Iran	RCT, I	No	Lumbar discectomy	Placebo	Morphine	105	44 (9)
					Pregabalin 300, NA, 300			40 (10)
					Pregabalin 300, NA, 300^b^			45 (12)
Vasigh et al,^41^ 2016	Iran	RCT, I	Yes	Lumbar laminectomy	Placebo	Morphine	114	50.2 (7.2)
					Gabapentin 600, NA, 300			49.5 (5.9)
					Gabapentin 300, NA, 300 plus Celecoxib 200, 200^b^			49.3 (6)
Qadeer et al,^42^ 2017	Pakistan	RCT, I	No	Lumbar discectomy	Pregabalin 150, NA, NA	Morphine	78	39 (12)
					Gabapentin 400, NA, NA			42 (8.9)
Yadav et al,^43^ 2018	India	RCT, I	No	Lumbar discectomy and laminectomy	Placebo	Fentanyl	60	41.6 (11.5)
					Pregabalin 150, NA, NA			43.8 (11.2
					Pregabalin 300, NA, NA			40.8 (11.0)
Urban et al,^44^ 2018	USA	RCT, I	Yes	Lumbar spinal fusion	Placebo	Hydromorphone plus morphine per os	86	56 (13)
					Pregabalin 150, NA, 150			57 (13)
Altiparmak et al,^45^ 2018	Turkey	RCT, I	No	Lumbar discectomy or laminectomy or spinal fusion	Placebo	Morphine	94	54 (11)
					Pregabalin 75, NA, 150			54 (11)
					Duloxetine 60, NA^b^			53 (11)
Routray et al,^46^ 2018	India	RCT, I	No	Lumbar discectomy	Placebo	Tramadol	75	39.76 (12.93)
					Pregabalin 300, NA, NA			36.56 (9.82)
					Gabapentin 600, NA, NA			35.36 (9.97)
Raja et al,^47^ 2019	India	RCT, I	Yes	Lumbar spinal fusion	Placebo	Morphine	97	51.6 (9.46)
					Pregabalin 75, NA, NA plus ketorolac 20, NA, NA			49.7 (12.33)
					plus acetaminophen 1000, NA, NA			
Momon et al,^48^ 2019	France	RCT, I	No	Lumbar discectomy or spinal fusion	Placebo	Oxycodone per os	145	41.8 (11.8)
					Pregabalin 150, NA, NA			41.1 (11.3)
					Dexamethasone 0.2/kg, NA, NA^b^			39.4 (10.9)
					Pregabalin 150, NA, NA plus Dexamethasone 0.2/kg, NA, NA^b^			41.5 (10.1)
Bala et al,^49^ 2019	India	RCT, I	No	Thoracolumbar laminectomy	Placebo	Fentanyl	75	39.76 (12.05)
					Pregabalin 150, NA, NA			31.72 (10.33)
					Clonidine 150, NA, NA^b^			34.08 (14.34)
Kien et al,^50^ 2019	Vietnam	RCT, I	No	Lumbar discectomy or laminectomy or spinal fusion	Placebo	Morphine	60	48.23 (11.88)
					Pregabalin 150, NA, NA plus Celecoxib 200, NA, NA			44.93 (10.26)
Zhang et al,^51^ 2021	China	RCT, I	Yes	Lumbar spinal fusion	Placebo	Tramadol plus flurbiprofen	93	55.5)
					Pregabalin 150, NA, 150 plus Celecoxib 400, NA, 400			59
					Ropivacaine 150^b^^,^^d^			59.5
Baloch et al,^24^ 2021	Pakistan	RCT, I	No	Lumbar discectomy	Placebo	Opioid^e^	84	NA (27- 61)
					Pregabalin 150, NA, 150			

Abbreviations: NA, not applicable; RCT, randomized clinical trial.

^a^ This table presents the key characteristics of the studies included in the systematic review and network meta-analysis. The table includes information such as the author(s), publication year, country, study design with level of evidence, detailed spine surgery, interventions (dosages of gabapentin and pregabalin), sample size, and other relevant details. The table provides a comprehensive overview of the included studies, allowing readers to examine the study characteristics and evaluate the relevance and quality of the evidence. Levels of evidence are defined according to Halperin et al.^23^ According to the Oxford Centre for Evidence-Based Medicine, level I, systematic review of randomized RCTs, denotes individual RCTs; level II, systemic review of cohort studies, denotes individual cohort studies and outcomes research; level III, systematic review of case-control studies, denotes individual case-control studies; level IV, case series (with or without comparison); and level V, expert opinion.

^b^ Not included in further analysis.

^c^ Refers to interquartile range.

^d^ Refers to subcutaneous infiltration at the end of surgery.

^e^ The study did not mention which opioid.

### Perioperative Outcomes

#### VAS Pain Score

This outcome included 20 trials with 1427 patients. According to the head-to-head comparisons (eFigure 3A in Supplement 1), all different dosages of gabapentin and pregabalin have lower VAS scores than placebo except for gabapentin 400 mg, 800 mg, and pregabalin 75 mg. Also, there was no significant difference between all different dosages of gabapentin and pregabalin. According to SUCRA probability (Table 2), gabapentin 900 mg (SUCRA, 90.8%; mean difference, −2.67%; 95% CI, −3.80% to −1.54%) was most likely to be ranked the best.

**Table 2.** SUCRA, Probability of Best, and Mean Rank of Different Dosages of Gabapentin and Pregabalin for Patients Undergoing Spinal Surgery^a^

VAS				Opioid consumption				Nausea			
Treatment	SUCRA, %	Probability of best, %	Mean rank	Treatment	SUCRA, %	Probability of best, %	Mean rank	Treatment	SUCRA, %	Probability of best, %	Mean rank
Gabapentin 900 mg	90.8	53.3	1.8	Gabapentin 900 mg	91.0	56.1	1.7	Pregabalin 150 mg	80.0	34.3	2.6
Gabapentin 1200 mg	86.6	26.1	2.2	Gabapentin 1200 mg	87.9	34.3	2.0	Pregabalin 300 mg	64.2	10.0	3.9
Gabapentin 600 mg	73.3	8.5	3.4	Gabapentin 600 mg	69.3|	3.9	3.5	Gabapentin 300 mg	63.6	20.9	3.9
Gabapentin 300 mg	57.7	3.2	4.8	Pregabalin 300 mg	63.1	3.1	4.0	Gabapentin 600 mg	63.1	10.7	4.0
Pregabalin 300 mg	48.9	1.1	5.6	Pregabalin 150 mg	46.9	0.2	5.2	Gabapentin 900 mg	52.6	14.4	4.8
Pregabalin 150 mg	45.4	0.0	5.9	Gabapentin 300 mg	31.2	0.2	6.5	Pregabalin 75 mg	36.0	0.9	6.1
Gabapentin 400 mg	34.1	3.4	6.9	Pregabalin 75 mg	25.3	0.8	7.0	Gabapentin 800 mg	35.0	7.8	6.2
Pregabalin 75 mg	33.4	0.2	7.0	Gabapentin 800 mg	20.8	1.4	7.3	Gabapentin 1200 mg	30.9	1.0	6.5
Gabapentin 800 mg	22.1	4.2	8.0	Placebo	14.5	0.0	7.8	Placebo	24.7	0.0	7.0
Placebo	7.7	0.0	9.3	NA	NA	NA	NA	NA	NA	NA	NA

Abbreviations: NA, not applicable; SUCRA, surface under the cumulative ranking curve; VAS, Visual Analog Scale.

^a^ The table presents the ranking for 3 different outcomes: VAS for pain intensity, opioid consumption, and nausea. The SUCRA values, presented as percentages, represent the probabilities of each treatment being the best treatment or one of the top-ranked treatments for the corresponding outcome. A higher SUCRA value indicates a higher likelihood that a treatment is among the top-ranked options. The probability of best values provide the probability of each treatment being the best treatment for the specific outcome. The closer the SUCRA and probability of best values are to 100%, the higher the probability that a therapy is the best treatment or one of the top-ranked treatments. The mean ranks indicate the relative effectiveness of each treatment option for each outcome, with lower ranks indicating better performance. The table provides a comprehensive overview of the comparative effectiveness of different dosages of gabapentin and pregabalin for pain control, opioid consumption, and nausea in patients undergoing spinal surgery.

#### Opioid Consumption

This outcome included 15 trials with 1070 patients. According to the head-to-head comparisons (eFigure 3B in Supplement 1), all different dosages of gabapentin and pregabalin have lower opioid consumption than placebo except for gabapentin 300 mg and 800 mg and pregabalin 75 mg. Also, gabapentin 900 mg and 1200 mg have lower opioid consumption than gabapentin 300 mg. According to SUCRA probability (Table 2), gabapentin 900 mg (SUCRA, 91.0%; mean difference, −22.07%; 95% CI, −33.22% to −10.92%) was most likely to be ranked the best.

#### Nausea

This outcome included 20 trials with 1388 patients. According to the head-to-head comparisons (eFigure 3C in Supplement 1), there is no significant difference between all different dosages of gabapentin and pregabalin. According to SUCRA probability (Table 2), pregabalin 150 mg (SUCRA, 80.0%; odds ratio, 0.41; 95% CI, 0.17-0.98) was most likely to be ranked the best. The results for vomiting and dizziness are included in eFigures 4, 5, and 6 in Supplement 1.

### Exploration for Inconsistency and Publication Bias

The study did not identify any inconsistencies in the outcomes using design-by-treatment interaction models. No significant imbalance was observed in the funnel plot, indicating no evidence of publication bias from small studies. Please refer to eTable 4, eFigure 7, and eFigure 8 in Supplement 1 for detailed results.

### CINeMA for Perioperative Outcomes

The study used a semiautomated web application, called CINeMA, to assess the confidence of network meta-analysis estimates for perioperative outcomes.^24^ The results of the certainty of evidence for each outcome can be found in eFigures 9 through 17 in Supplement 1.

### Network Meta-Regression Analysis

In our study, we conducted a meta-regression analysis to assess the study-level characteristics of comedication, pharmaceutical funding, postoperative gabapentinoid use and preexisting neuropathic pain on the treatment effects. Our analysis revealed that these factors had no significant association with the outcomes of interest except that funding may be associated with the outcome of nausea. These findings suggest that the use of additional pain medications during the perioperative period, postoperative gabapentinoid use, and preexisting neuropathic pain did not substantially impact the reported pain intensity or opioid consumption in our study population (eTables 5 and 6 in Supplement 1). We further conducted a post hoc analysis for the results of nausea by excluding studies influenced by funding (eTables 7 and 8 and eFigure 13 in Supplement 1).

## Discussion

In this systematic review and network meta-analysis, we evaluated the associations of pain, opioid consumption, and adverse events with different dosages of pregabalin and gabapentin in patients undergoing spine surgery. We analyzed data from 27 RCTs with 1861 patients and found that gabapentin 900 mg per day was associated with the lowest VAS pain score among all dosages. Additionally, we found no differences in adverse events (nausea, vomiting, and dizziness) among all treatments. These results suggest that gabapentin 900 mg per day before spine surgery may be an effective and safe option for reducing pain and opioid consumption in these patients.

Canavan et al^52^ conducted a systematic review and meta-analysis that evaluated the effectiveness, adverse events, and withdrawal rates of various pharmacological interventions for managing chronic spinal cord injury pain. They found that tricyclic antidepressants, gabapentinoids, and opioids were the most effective in reducing pain, with the lowest withdrawal rates. They also found that these interventions had common adverse events, but they were generally mild to moderate. Tong et al^6^ conducted a network meta-analysis with 7 trials and found that pregabalin was the most effective for relieving pain and gabapentin performed better in aspects associated with drug therapy–related safety for patients with spinal cord injury. A Cochrane review conducted by Wiffen et al^53^ examined the existing research on the use of gabapentin for managing chronic neuropathic pain in adults. The study found that gabapentin at doses of 1200 mg or more daily can provide good levels of pain relief for some people with postherpetic neuralgia and painful diabetic neuropathy. The study also found that adverse events withdrawals were more common with gabapentin than with placebo and that individual adverse events occurred significantly more often with gabapentin, such as dizziness, somnolence, peripheral edema, and gait disturbance. Although those studies focused on neurogenic pain, our study targeted a different subset of population. Our study specifically focuses on pregabalin and gabapentin in patients undergoing spine surgery, whereas Tong et al^6^ and Canavan et al^52^ evaluated a wide range of pharmacological interventions for chronic spinal cord injury pain, and Wiffen et al^53^ focused on postherpetic neuralgia and painful diabetic neuropathy.

Martinez et al^54^ evaluated the effectiveness of perioperative pregabalin administration in preventing chronic postoperative pain across different surgical procedures. They found that perioperative pregabalin administration does not prevent chronic postoperative pain, whereas our study found that pregabalin may have benefits in reducing pain in spine surgery. Verret et al^55^ conducted a large meta-analysis of 281 trials and assessed the effectiveness and safety of gabapentinoids in reducing postoperative pain and reducing opioid consumption after surgery. They concluded that there is no clinically significant analgesic effect for the perioperative use of gabapentinoids and also no effect on the prevention of postoperative chronic pain, with more risk of adverse events. Our study focused on patients undergoing spine surgery and found that gabapentin 900 mg per day before spine surgery was associated with the lowest VAS pain score among all dosages, and no differences in adverse events were noted among all treatments.

Optimal pain control for spine surgery necessitates a multimodal approach, as emphasized by the guideline recommendations put forth by Peene et al^56^ and Waelkens et al.^57^ These studies underscore the significance of adopting a comprehensive strategy that combines various interventions to effectively manage pain and enhance patient outcomes. Peene et al^56^ noted that although gabapentinoids have proven efficacy in managing pain in the patient population undergoing lumbar laminectomy, they are not recommended as the first line of treatment because of the substantial risk of important adverse effects, including sedation, dizziness, and visual blurring. It is important to consider the risk-benefit profile of gabapentinoids and exercise caution when prescribing them. Our study further contributes to this understanding by specifically examining the comparative effectiveness of different dosages of gabapentinoids in the context of spine surgery, providing additional insights into their use and helping clinicians make informed decisions regarding pain management strategies in this population. In contrast to the study by Waelkens et al,^57^ which concluded that gabapentinoids are not recommended as part of a multimodal analgesic regimen in complex spine surgery because of limited evidence and concerns regarding adverse effects. Our study found no significant difference in the occurrence of adverse effects when comparing different dosages of gabapentinoids in patients undergoing spine surgery. This suggests that, within the dosages evaluated, the use of gabapentinoids does not pose a greater risk of adverse effects compared with other pain medications. However, it is important to note that the potential for adverse effects, such as sedation and respiratory depression, is still a concern with gabapentinoid use, as highlighted in the studies by Peene et al^56^ and Waelkens et al.^57^ Therefore, clinicians should carefully consider the risk-benefit profile of gabapentinoids and monitor patients closely for any adverse reactions when using these medications in the perioperative period. Our study adds to the existing literature by providing specific insights into the comparative effectiveness and safety of gabapentinoids in pain management during spine surgery, helping clinicians make informed decisions regarding their use in this context.

The use of gabapentinoids, such as gabapentin, to reduce opioid consumption in perioperative pain management has important implications. By minimizing opioid use, we can improve patient outcomes, mitigate risks and complications, and contribute to efforts to address the opioid crisis. Our study adds to the growing evidence supporting multimodal analgesic approaches that combine nonopioid medications and techniques for optimal pain control while minimizing opioid requirements. Reducing opioid consumption should be a primary goal in perioperative pain management, and our study highlights the potential of gabapentinoids, specifically gabapentin, in achieving this goal.

In the context of our study and the findings from the 2005 study by Pandey et al,^27^ it is important to discuss the differences observed between gabapentin 900 mg and 600 mg. The study by Pandey et al^27^ did not find gabapentin 900 mg to be superior to 600 mg in terms of pain control, which contrasts with our network meta-analysis results where gabapentin 900 mg demonstrated a lower VAS score compared with placebo and compared with gabapentin 600 mg. Several factors may explain these divergent findings. Variations in patient characteristics, surgical procedures, or study methods between the Pandey study^27^ and the trials included in our meta-analysis could contribute to the disparate outcomes. It is important to note that network meta-analysis offers a more comprehensive synthesis of evidence by incorporating both direct and indirect treatment comparisons. This approach allows for a more reliable estimation of treatment effects, particularly when there is a lack of head-to-head trials comparing specific dosages. However, despite the favorable findings for gabapentin 900 mg in our network meta-analysis, the presence of large overlap in the confidence intervals for the VAS estimates between gabapentin 900 mg and 600 mg suggests some uncertainty in the precise magnitude of the difference. In our study, we aimed to investigate the potential confounding effect of perioperative comedication and pharmaceutical funding on the primary outcomes of pain intensity, secondary outcomes of opioid consumption, and adverse effects. To address this, we performed a meta-regression analysis that allowed us to examine the influence of comedication use and funding source on the study outcomes. Specifically, we analyzed the impact of commonly used painkillers such as acetaminophen, celecoxib, and ketorolac, as well as the presence of funding from pharmaceutical companies or other foundations. Interestingly, our analysis revealed that neither the use of comedication nor the funding source had a significant association with the reported pain intensity, opioid consumption, or occurrence of adverse effects in our study population. These findings indicate that the inclusion of additional pain medications during the perioperative period and the funding source of the studies did not have a substantial impact on the observed outcomes. By conducting this meta-regression analysis, we provided more robust and reliable findings. It is important to consider these results when interpreting the overall findings of our study and when designing future research studies in the field of perioperative pain management.

### Limitations

Although, to our knowledge, this study is the first network meta-analysis on this topic, it has several limitations. The included studies exhibited heterogeneity in methods and patient populations, which may impact the overall conclusions. The limited number of subjects receiving gabapentin 900 mg per day reduced the precision of our estimates for this dosage. The heterogeneity in the duration of perioperative administration of gabapentinoids could introduce variability in treatment effects. Our study primarily focused on short-term outcomes and may not capture long-term effectiveness and safety data. Adverse events not included in the study may exist owing to the lack of comprehensive data. The generalizability of our findings to all patients undergoing spine surgery may be limited because of differences in surgical procedures and patient populations among the included trials. The determination of MCID for postoperative pain, particularly in the context of spine surgery, is not straightforward and can vary depending on multiple factors such as the patient population, the surgical procedure, and the measurement instrument used. This limitation restricts the accuracy and interpretation of the SUCRA values, which should be interpreted cautiously. Future research with larger sample sizes, robust methods, standardized outcome measures, and longer follow-up durations is needed to address these limitations and provide more conclusive evidence on the optimal dosages and safety of gabapentinoids in the perioperative setting.

## Conclusions

In conclusion, our study findings suggest that the preoperative administration of gabapentin and pregabalin may effectively alleviate postoperative pain and reduce opioid consumption in patients undergoing spine surgery. However, it is important to note that increasing the dosage of gabapentin above 900 mg was not found to be associated with further pain reduction. It is crucial for future research to encompass larger, well-designed RCTs to validate our findings and ascertain the optimal dosage and long-term safety of these medications for patients undergoing spine surgery.

## Notes

Supplement 1. **eFigure 1.** Network Graph for Vomit and Dizziness **eFigure 2.** Risk of Bias Assessment of the Included Studies **eFigure 3.** Network Meta-Analysis League Tables **eFigure 4.** Rank Plot **eFigure 5.** Forest Plot of Network Meta-Analysis Results (Placebo as Reference) **eFigure 6.** Forest Plot of Pairwise Comparison for Each Outcome **eFigure 7.** Publication Bias: Funnel Plot **eFigure 8.** Publication Bias: The Egger Test **eFigure 9.** CINeMA for the Perioperative Outcomes (VAS, Opioid Consumption, Nausea, Vomit, Dizziness) **eTable 1.** Electronic Database Search Strategy **eTable 2.** Excluded Studies With Reasons **eTable 3.** Descriptions and Demo of the Included Studies **eTable 4.** Assessment of Inconsistency With Design-by-Treatment Interaction Models **eTable 5.** Meta-Regression of Covariates for Outcomes **eTable 6.** SUCRA, PrBest, and Mean Rank of Different Dosages of Gabapentin and Pregabalin for Patients Undergoing Spinal Surgery **eFigure 10.** Network Graph for Nausea Without Funding **eFigure 11.** Network Meta-Analysis League Tables for Nausea Without Funding **eFigure 12.** Rank Plot for Nausea Without Funding **eFigure 13.** Forest Plot of Network Meta-Analysis Results for Nausea Without Funding (Placebo as Reference) **eFigure 14.** Forest Plot of Pairwise Comparison for Nausea Without Funding **eFigure 15.** Publication Bias: Funnel Plot for Nausea Without Funding **eFigure 16.** Publication Bias: The Egger Test for Nausea Without Funding **eFigure 17.** CINeMA for Nausea Without Funding **eTable 7.** SUCRA, PrBest, and Mean Rank of Different Dosages of Gabapentin and Pregabalin for Patients Undergoing Spinal Surgery **eTable 8.** Assessment of Inconsistency With Design-by-Treatment Interaction Models
eReferences

Supplement 2.
Data Sharing Statement
