# Supplementary material for: Different Gabapentin and Pregabalin Dosages for Perioperative Pain Control in Patients Undergoing Spine Surgery: A Systematic Review and Network Meta-Analysis
Source: JAMA Netw Open. 2023 Aug 9;6(8):e2328121. doi: 10.1001/jamanetworkopen.2023.28121 (PMC10413173; doi:10.1001/jamanetworkopen.2023.28121)
Supplement: Supplement 1. — eFigure 1. Network Graph for Vomit and Dizziness eFigure 2. Risk of Bias Assessment of the Included Studies eFigure 3. Network Meta-Analysis League Tables eFigure 4. Rank Plot eFigure 5. Forest Plot of Network Meta-Analysis Results (Placebo as Reference) eFigure 6. Forest Plot of Pairwise Comparison for Each Outcome eFigure 7. Publication Bias: Funnel Plot eFigure 8. Publication Bias: The Egger Test eFigure 9. CINeMA for the Perioperative Outcomes (VAS, Opioid Consumption, Nausea, Vomit, Dizziness) eTable 1. Electronic Database Search Strategy eTable 2. Excluded Studies With Reasons eTable 3. Descriptions and Demo of the Included Studies eTable 4. Assessment of Inconsistency With Design-by-Treatment Interaction Models eTable 5. Meta-Regression of Covariates for Outcomes eTable 6. SUCRA, PrBest, and Mean Rank of Different Dosages of Gabapentin and Pregabalin for Patients Undergoing Spinal Surgery eFigure 10. Network Graph for Nausea Without Funding eFigure 11. Network Meta-Analysis League Tables for Nausea Without Funding eFigure 12. Rank Plot for Nausea Without Funding eFigure 13. Forest Plot of Network Meta-Analysis Results for Nausea Without Funding (Placebo as Reference) eFigure 14. Forest Plot of Pairwise Comparison for Nausea Without Funding eFigure 15. Publication Bias: Funnel Plot for Nausea Without Funding eFigure 16. Publication Bias: The Egger Test for Nausea Without Funding eFigure 17. CINeMA for Nausea Without Funding eTable 7. SUCRA, PrBest, and Mean Rank of Different Dosages of Gabapentin and Pregabalin for Patients Undergoing Spinal Surgery eTable 8. Assessment of Inconsistency With Design-by-Treatment Interaction Models eReferences [file jamanetwopen-e2328121-s001.pdf]

Supplemental Online Content

Tsai SHL, Hu CW, El Sammak S, et al. Different gabapentin and pregabalin dosages for perioperative pain control in patients undergoing spine surgery: a systematic review and network meta-analysis. *JAMA Netw Open*. 2023;6(8):e2328121. doi:10.1001/jamanetworkopen.2023.28121

eFigure 1. Network Graph for Vomit and Dizziness
eFigure 2. Risk of Bias Assessment of the Included Studies
eFigure 3. Network Meta-Analysis League Tables
eFigure 4. Rank Plot
eFigure 5. Forest Plot of Network Meta-Analysis Results (Placebo as Reference)
eFigure 6. Forest Plot of Pairwise Comparison for Each Outcome
eFigure 7. Publication Bias: Funnel Plot
eFigure 8. Publication Bias: The Egger Test
eFigure 9. CINeMA for the Perioperative Outcomes (VAS, Opioid Consumption, Nausea, Vomit, Dizziness)
eTable 1. Electronic Database Search Strategy
eTable 2. Excluded Studies With Reasons
eTable 3. Descriptions and Demo of the Included Studies
eTable 4. Assessment of Inconsistency With Design-by-Treatment Interaction Models
eTable 5. Meta-Regression of Covariates for Outcomes
eTable 6. SUCRA, PrBest, and Mean Rank of Different Dosages of Gabapentin and Pregabalin for Patients Undergoing Spinal Surgery
eFigure 10. Network Graph for Nausea Without Funding
eFigure 11. Network Meta-Analysis League Tables for Nausea Without Funding
eFigure 12. Rank Plot for Nausea Without Funding
eFigure 13. Forest Plot of Network Meta-Analysis Results for Nausea Without Funding (Placebo as Reference)
eFigure 14. Forest Plot of Pairwise Comparison for Nausea Without Funding
eFigure 15. Publication Bias: Funnel Plot for Nausea Without Funding
eFigure 16. Publication Bias: The Egger Test for Nausea Without Funding
eFigure 17. CINeMA for Nausea Without Funding
eTable 7. SUCRA, PrBest, and Mean Rank of Different Dosages of Gabapentin and Pregabalin for Patients Undergoing Spinal Surgery
eTable 8. Assessment of Inconsistency With Design-by-Treatment Interaction Models
eReferences

This supplemental material has been provided by the authors to give readers additional information about their work.

© 2023 Tsai SHL et al. *JAMA Network Open.*

eFigure 1: Network Graph for Vvomit and Dizziness

* The sizes of the nodes are proportional to the included cases randomized to the treatments, and the number on the lines indicate the number of studies comparing two nodes.

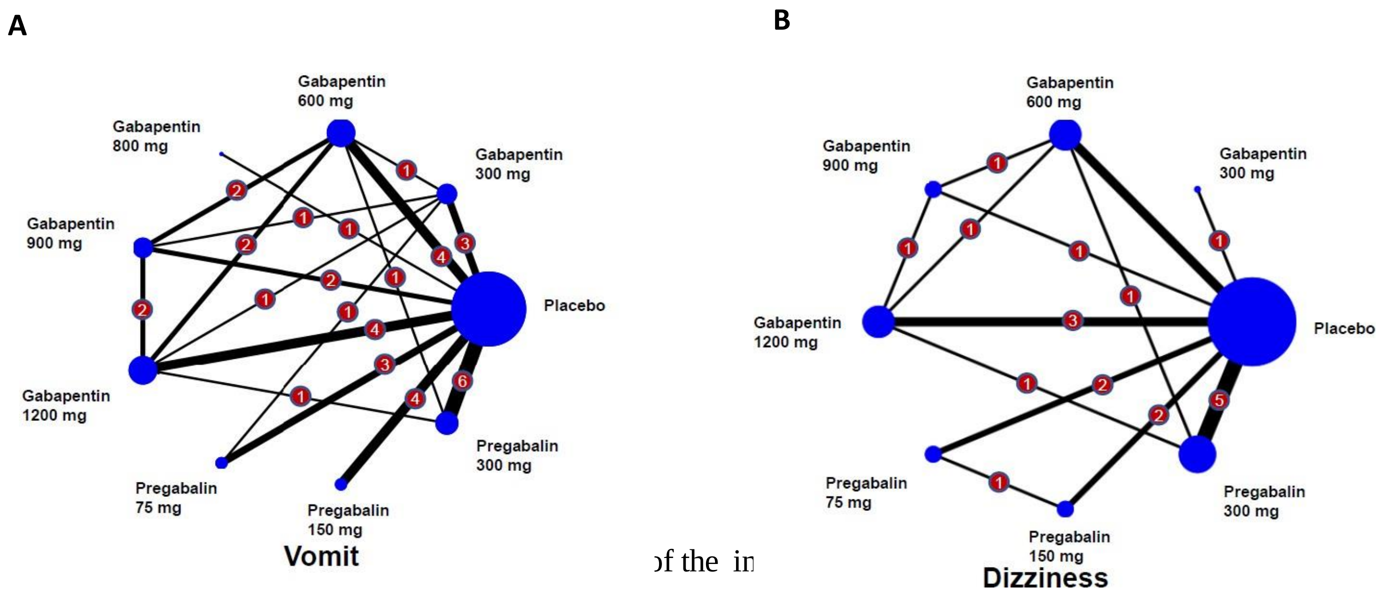

eFigure 2 Risk of Bias Assessment of the Included Studies

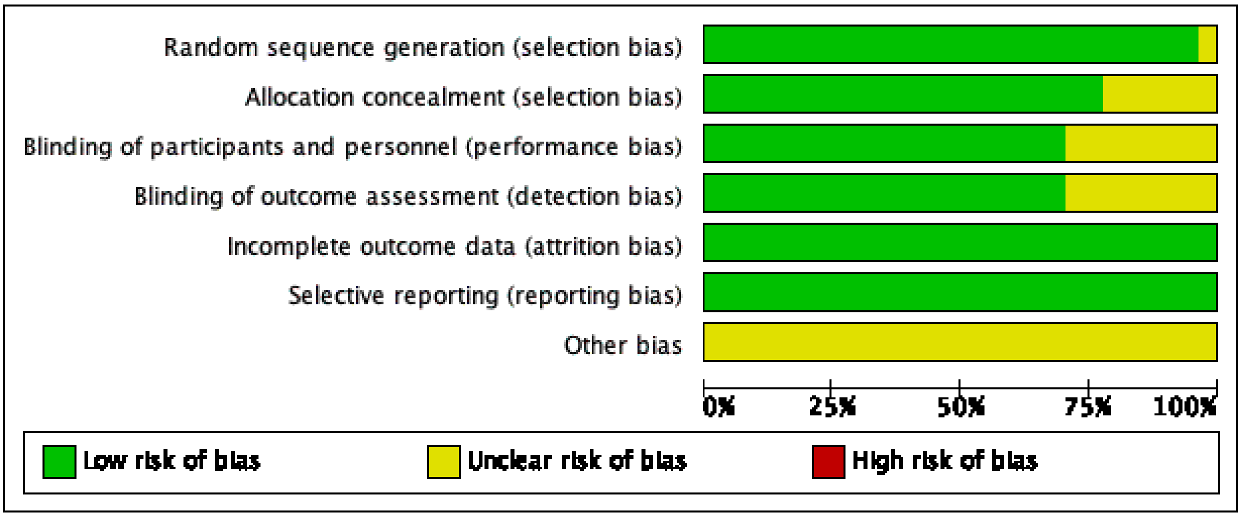

© 2023 Tsai SHL et al. *JAMA Network Open.*

	Random sequence generation (selection bias)	Allocation concealment (selection bias)	Blinding of participants and personnel (performance bias)	Blinding of outcome assessment (detection bias)	Incomplete outcome data (attrition bias)	Selective reporting (reporting bias)	Other bias
Altiparmak 2018	+	+	+	+	+	+	?
Bala 2019	+	+	?	?	+	+	?
Baloch 2021	?	+	+	+	+	+	?
Burke 2010	+	+	+	?	+	+	?
Choi 2013	+	+	+	+	+	+	?
Gianesello 2012	+	+	+	+	+	+	?
Hegarty 2011	+	+	+	+	+	+	?
Khan 2011	+	?	+	+	+	+	?
Khurana 2014	+	+	?	+	+	+	?
Kien 2019	+	+	?	?	+	+	?
Kim 2011	+	+	+	+	+	+	?
Kumar 2013	+	+	+	+	+	+	?
Momon 2019	+	+	+	+	+	+	?
Ozgencil 2011	+	+	?	?	+	+	?
Pandey 2004	+	+	+	+	+	+	?
Pandey 2005	+	?	+	+	+	+	?
Qadeer 2017	+	+	?	?	+	+	?
Radhakrishnan 2005	+	+	+	+	+	+	?
Raja 2019	+	+	?	+	+	+	?
Routray 2018	+	+	?	?	+	+	?
Spreng 2011	+	?	+	?	+	+	?
Turan 2004	+	+	+	+	+	+	?
Urban 2018	+	?	+	+	+	+	?
Vasigh 2016	+	+	+	+	+	+	?
Yadav 2018	+	?	+	+	+	+	?
Zarei 2016	+	?	+	+	+	+	?
Zhang 2021	+	+	?	?	+	+	?

© 2023 Tsai SHL et al. *JAMA Network Open.*

eFigure 3: Network Meta-Analysis League Tables

(Comparisons between treatments (column vs row) should be read from left to right and the order of the treatments in the diagonal doesn't reflect ranking)

(*: Significant difference)

A: Network meta-analysis league tables for postoperative pain intensity (VAS)
(In the left lower half, mean differences lower than 0 favor the row-defining treatment)

Placebo									
*1.64 (0.42,2.86)	Gabapentin 300mg								
0.94 (-0.90,2.78)	-0.70 (-2.91,1.50)	Gabapentin 400mg							
*2.11 (1.12,3.10)	0.47 (-0.93,1.87)	1.17 (-0.91,3.26)	Gabapentin 600mg						
0.00 (-3.27,3.27)	-1.64 (-5.14,1.85)	-0.94 (-4.69,2.82)	-2.11 (-5.53,1.31)	Gabapentin 800mg					
*2.67 (1.54,3.80)	1.03 (-0.41,2.47)	1.74 (-0.42,3.89)	0.56 (-0.66,1.79)	2.67 (-0.79,6.14)	Gabapentin 900mg				
*2.49 (1.58,3.39)	0.84 (-0.51,2.20)	1.55 (-0.50,3.60)	0.38 (-0.75,1.50)	2.49 (-0.91,5.88)	-0.19 (-1.37,1.00)	Gabapentin 1200mg			
0.94 (-0.05,1.93)	-0.70 (-2.27,0.87)	0.00 (-2.05,2.05)	-1.17 (-2.57,0.23)	0.94 (-2.48,4.36)	-1.73 (-3.24,-0.23)	-1.55 (-2.89,-0.21)	Pregabalin 75mg		
*1.31 (0.6,1.99)	-0.33 (-1.73,1.07)	0.37 (-1.34,2.08)	-0.80 (-2.00,0.40)	1.31 (-2.04,4.65)	-1.37 (-2.69,-0.04)	-1.18 (-2.31,-0.05)	0.37 (-0.77,1.50)	Pregabalin 150mg	
*1.41 (0.41,2.41)	-0.23 (-1.78,1.31)	0.47 (-1.62,2.56)	-0.70 (-2.06,0.66)	1.41 (-2.01,4.83)	-1.26 (-2.72,0.19)	-1.08 (-2.28,0.13)	0.47 (-0.93,1.88)	0.10 (-1.10,1.31)	Pregabalin 300mg

© 2023 Tsai SHL et al. *JAMA Network Open.*

B: Network meta-analytic relative treatment effects for opioid consumption
(In the left lower half, mean differences lower than 0 favor the row-defining treatment)

Placebo								
-4.24 (-15.87,7.40)	Gabapentin 300mg							
*-15.25 (-23.48,-7.02)	-11.01 (-23.93,1.91)	Gabapentin 600mg						
0.40 (-17.56,18.36)	4.64 (-16.76,26.03)	15.65 (-4.11,35.41)	Gabapentin 800mg					
*-22.07 (-33.22,-10.92)	*-17.83 (-32.11,-3.56)	-6.82 (-18.44,4.80)	*-22.47 (-43.61,-1.33)	Gabapentin 900mg				
*-20.36 (-28.80,-11.93)	*-16.13 (-29.18,-3.08)	-5.12 (-15.13,4.90)	*-20.76 (-40.61,-0.92)	1.70 (-10.02,13.43)	Gabapentin 1200mg			
-1.92 (-19.27,15.43)	2.32 (-18.57,23.20)	13.33 (-5.87,32.53)	-2.32 (-27.29,22.65)	20.15 (-0.47,40.77)	18.44 (-0.85,37.73)	Pregabalin 75mg		
*-9.33 (-17.90,-0.75)	-5.09 (-19.47,9.28)	5.92 (-5.77,17.61)	-9.73 (-29.63,10.17)	12.74 (-1.19,26.67)	11.04 (-0.79,22.87)	-7.41 (-26.76,11.94)	Pregabalin 150mg	
*-13.60 (-21.74,-5.47)	-9.37 (-23.21,4.47)	1.64 (-8.84,12.12)	-14.00 (-33.72,5.71)	8.46 (-4.76,21.68)	6.76 (-3.88,17.40)	-11.68 (-30.85,7.48)	-4.28 (-15.19,6.63)	Pregabalin 300mg

© 2023 Tsai SHL et al. *JAMA Network Open.*

C: Network meta-analytic relative treatment effects for Nausea
(In the left lower half, odds ratio lower than 1 favor the row-defining treatment)

Placebo								
0.57 (0.17,1.85)	Gabapentin 300mg							
0.57 (0.23,1.41)	1.01 (0.24,4.31)	Gabapentin 600mg						
1.00 (0.22,4.63)	1.76 (0.25,12.19)	1.74 (0.29,10.27)	Gabapentin 800mg					
0.66 (0.16,2.72)	1.16 (0.19,6.97)	1.15 (0.27,4.90)	0.66 (0.08,5.31)	Gabapentin 900mg				
0.95 (0.41,2.19)	1.68 (0.41,6.85)	1.66 (0.55,4.98)	0.95 (0.17,5.46)	1.45 (0.33,6.30)	Gabapentin 1200mg			
0.88 (0.39,2.00)	1.55 (0.42,5.70)	1.53 (0.47,5.05)	0.88 (0.15,5.02)	1.34 (0.26,6.76)	0.92 (0.29,2.95)	Pregabalin 75mg		
*0.41 (0.17,0.98)	0.72 (0.17,3.09)	0.71 (0.20,2.47)	0.41 (0.07,2.39)	0.62 (0.12,3.26)	0.43 (0.13,1.43)	0.46 (0.15,1.46)	Pregabalin 150mg	
0.57 (0.27,1.21)	1.01 (0.25,4.05)	0.99 (0.33,2.99)	0.57 (0.10,3.16)	0.87 0.18,4.14)	0.60 (0.22,1.65)	0.65 (0.21,1.98)	1.40 (0.44,4.47)	Pregabalin 300mg

© 2023 Tsai SHL et al. *JAMA Network Open.*

D: Network meta-analytic relative treatment effects for Vomit
(In the left lower half, odds ratio lower than 1 favor the row-defining treatment)

Placebo								
0.87 (0.32,2.41)	Gabapentin 300mg							
*0.47 (0.22,0.99)	0.53 (0.16,1.76)	Gabapentin 600mg						
0.64 (0.10,4.15)	0.74 (0.09,6.15)	1.38 (0.18,10.30)	Gabapentin 800mg					
0.35 (0.07,1.73)	0.40 (0.07,2.39)	0.74 (0.15,3.78)	0.54 (0.05,6.33)	Gabapentin 900mg				
0.45 (0.18,1.13)	0.51 (0.14,1.86)	0.96 (0.33,2.83)	0.70 (0.09,5.61)	1.30 (0.24,7.08)	Gabapentin 1200mg			
0.58 (0.22,1.52)	0.66 (0.17,2.61)	1.24 (0.37,4.20)	0.90 (0.11,7.35)	1.67 (0.26,10.83)	1.29 (0.34,4.87)	Pregabalin 75mg		
0.39 (0.14,1.11)	0.44 (0.10,1.91)	0.83 (0.23,3.02)	0.60 (0.07,5.12)	1.12 (0.16,7.62)	0.86 (0.21,3.48)	0.67 (0.16,2.78)	Pregabalin 150mg	
0.57 (0.30,1.10)	0.65 (0.20,2.15)	1.22 (0.49,3.07)	0.89 (0.12,6.41)	1.65 (0.30,9.06)	1.27 (0.44,3.64)	0.99 (0.31,3.15)	1.47 (0.43,5.09)	Pregabalin 300mg

© 2023 Tsai SHL et al. *JAMA Network Open.*

E: Network meta-analytic relative treatment effects for Dizziness
(In the left lower half, odds ratio lower than 1 favor the row-defining treatment)

Placebo							
0.32 (0.01,8.24)	Gabapentin 300mg						
1.52 (0.70,3.30)	4.72 (0.17,132.59)	Gabapentin 600mg					
1.17 (0.17,8.31)	3.65 (0.08,161.20)	0.77 (0.10,6.05)	Gabapentin 900mg				
1.66 (0.77,3.62)	5.17 (0.18,145.26)	1.10 (0.38,3.13)	1.42 (0.20,9.88)	Gabapentin 1200mg			
1.95 (0.43,8.86)	6.05 (0.17,217.05)	1.28 (0.23,7.04)	1.66 (0.14,19.72)	1.17 (0.21,6.42)	Pregabalin 75mg		
0.85 (0.21,3.42)	2.66 (0.08,90.42)	0.56 (0.11,2.76)	0.73 (0.07,8.02)	0.51 (0.10,2.51)	0.44 (0.09,2.13)	Pregabalin 150mg	
1.12 (0.57,2.19)	3.48 (0.13,95.59)	0.74 (0.29,1.88)	0.96 (0.13,7.24)	0.67 (0.28,1.61)	0.58 (0.11,3.02)	1.31 (0.28,6.11)	Pregabalin 300mg

© 2023 Tsai SHL et al. *JAMA Network Open.*

eFigure 4: Rank Plot

A: VAS

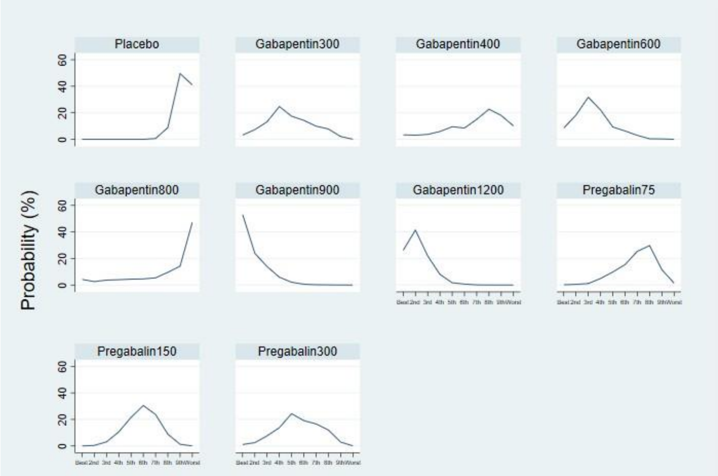

B: Opioid consumption

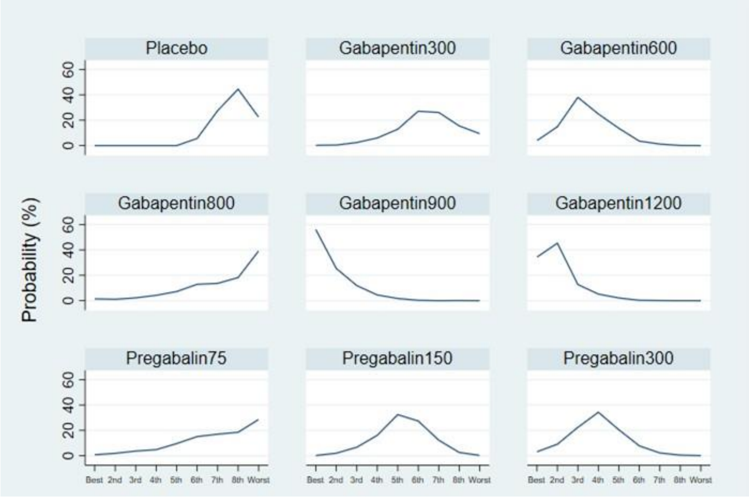

C: Nausea

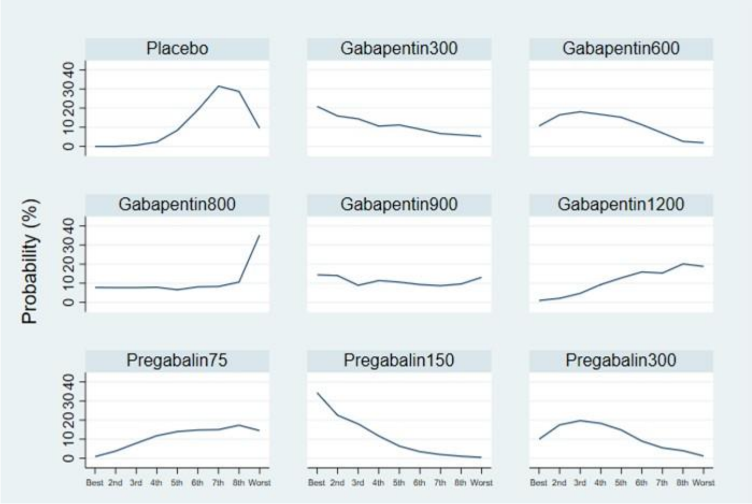

D: Vomit

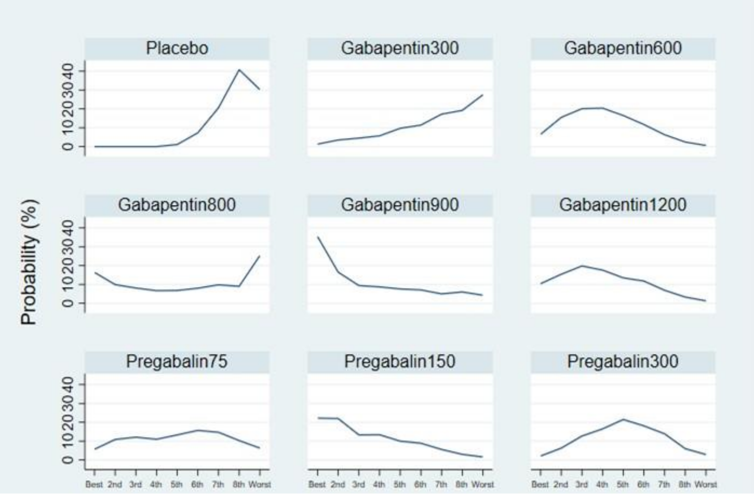

© 2023 Tsai SHL et al. *JAMA Network Open*.

E: Dizziness

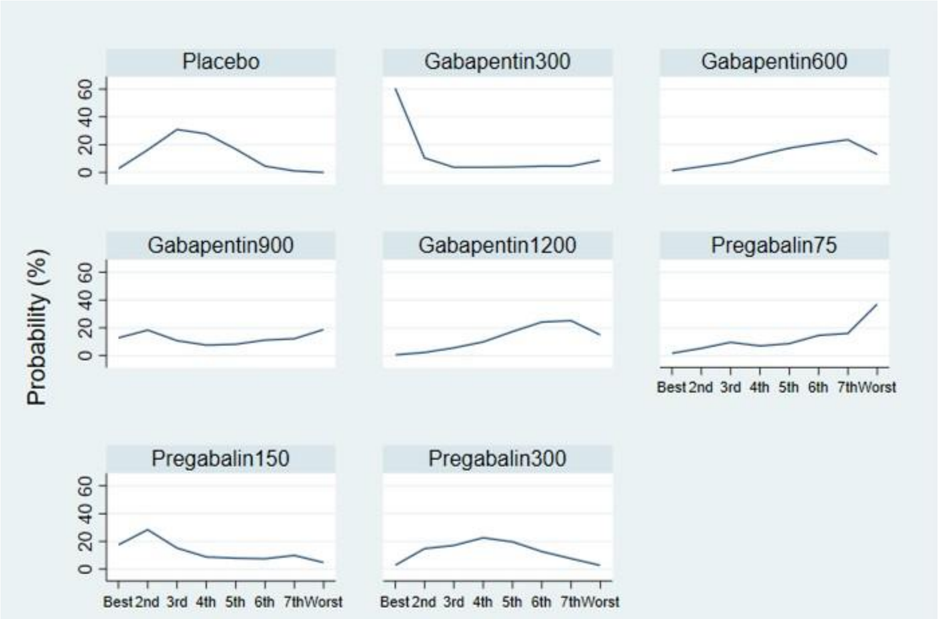

© 2023 Tsai SHL et al. *JAMA Network Open.*

eFigure 5: Forest Plot of Network Meta-Analysis Results (Placebo as Reference)

A: VAS

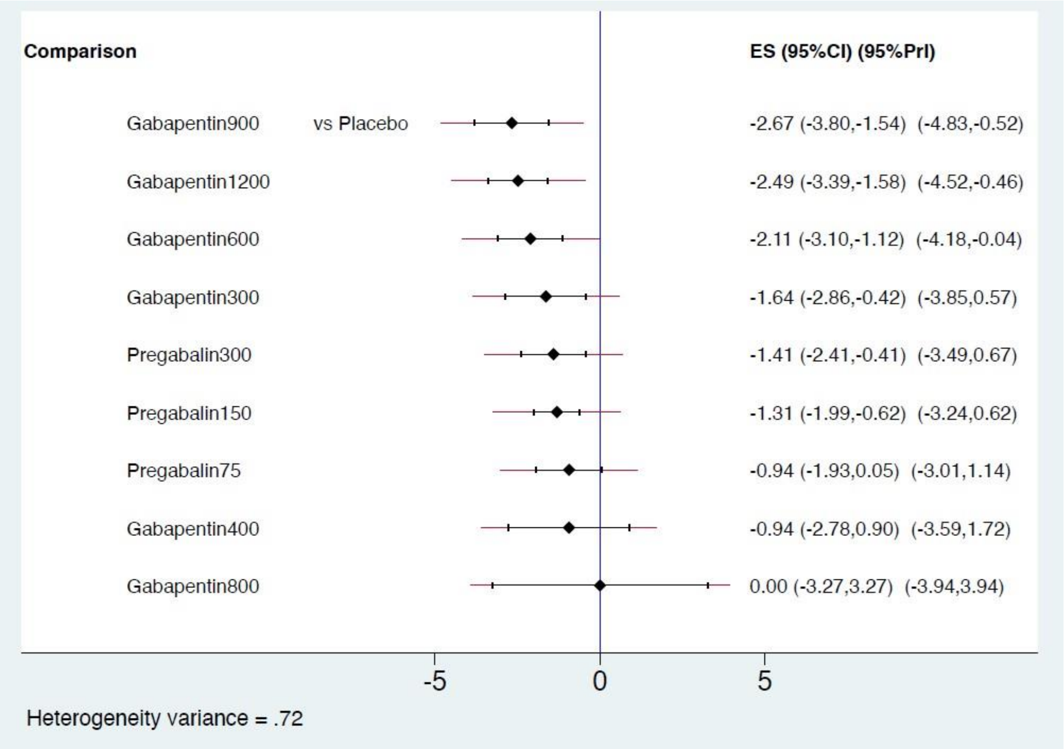

B: Opioid consumption

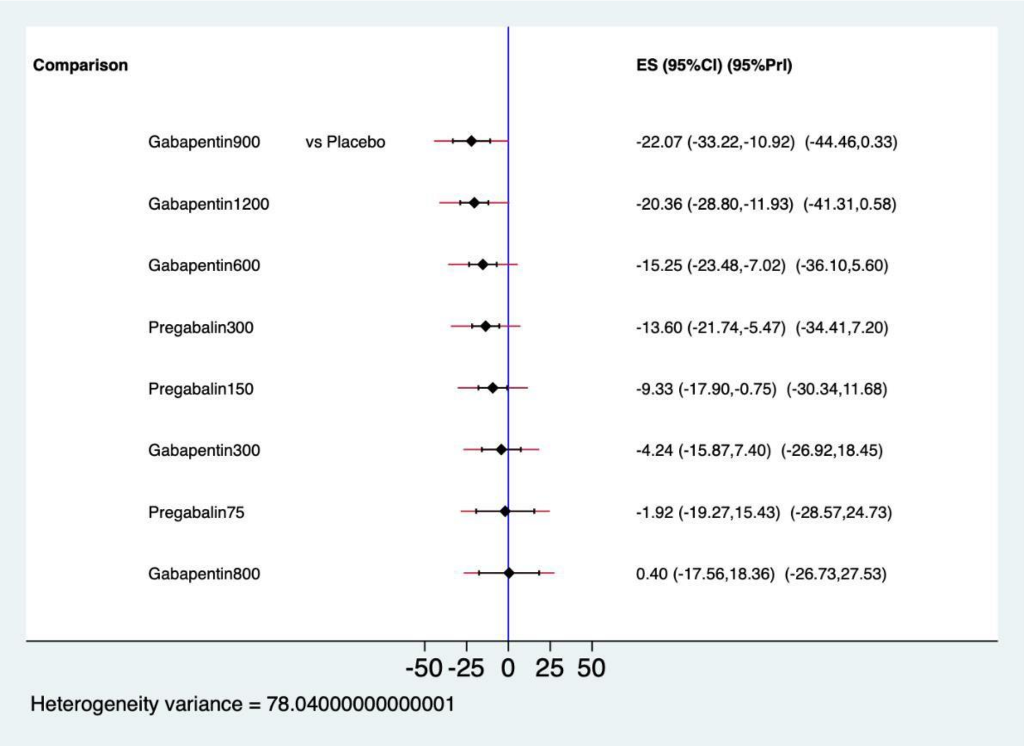

© 2023 Tsai SHL et al. *JAMA Network Open.*

C: Nausea

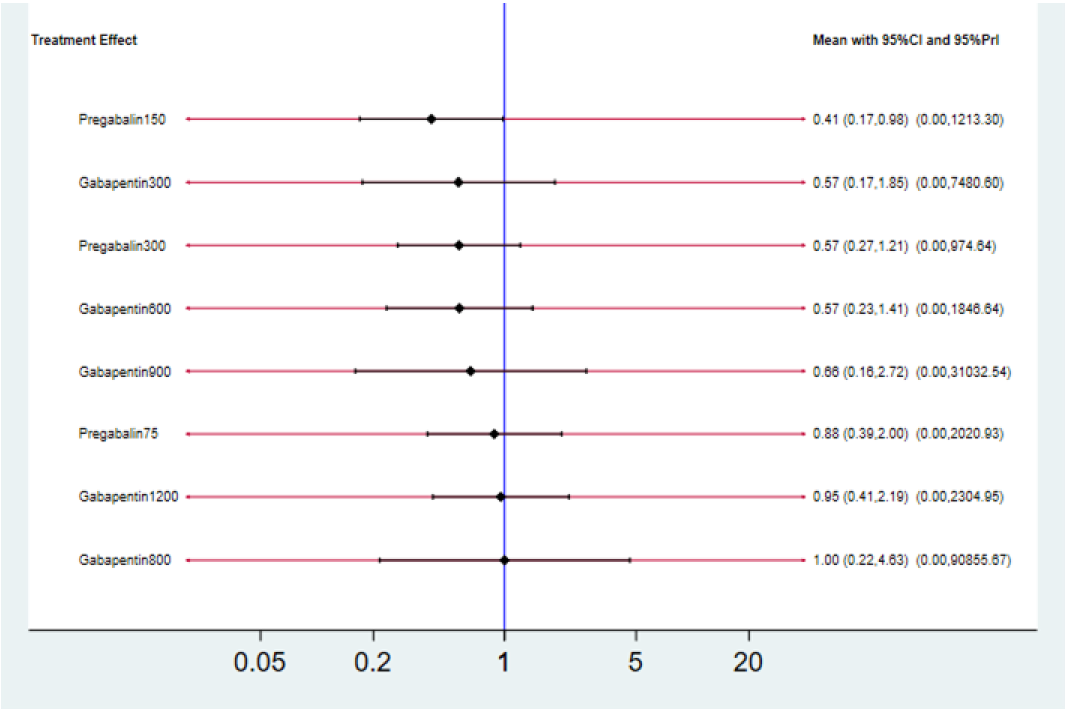

D: Vomit

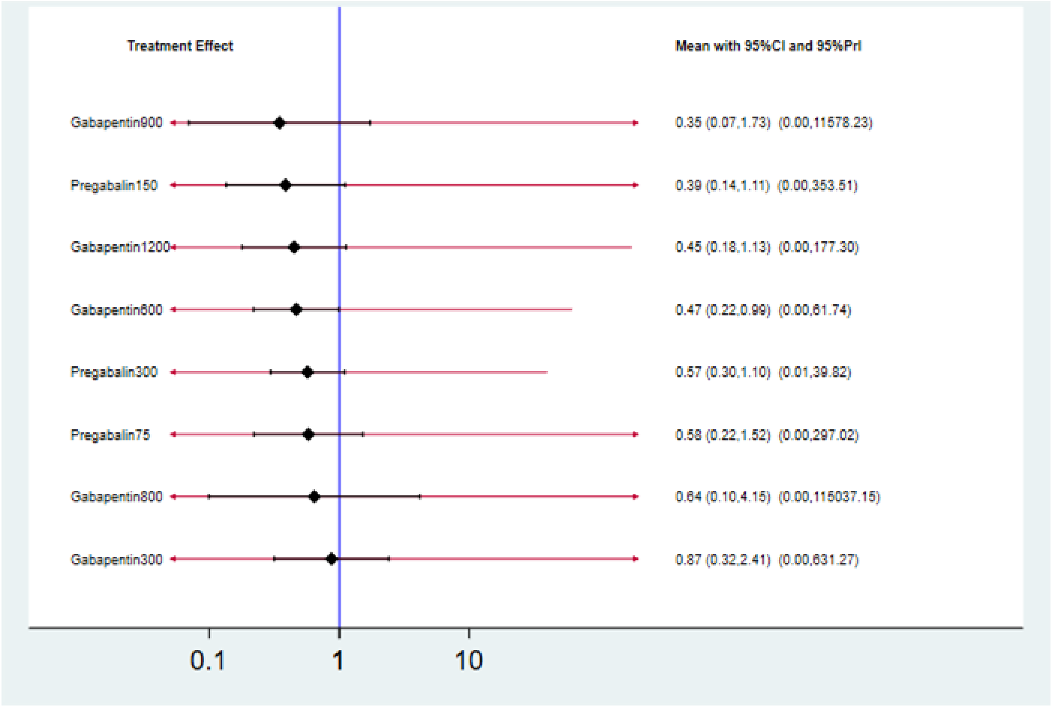

© 2023 Tsai SHL et al. *JAMA Network Open.*

E: Dizziness

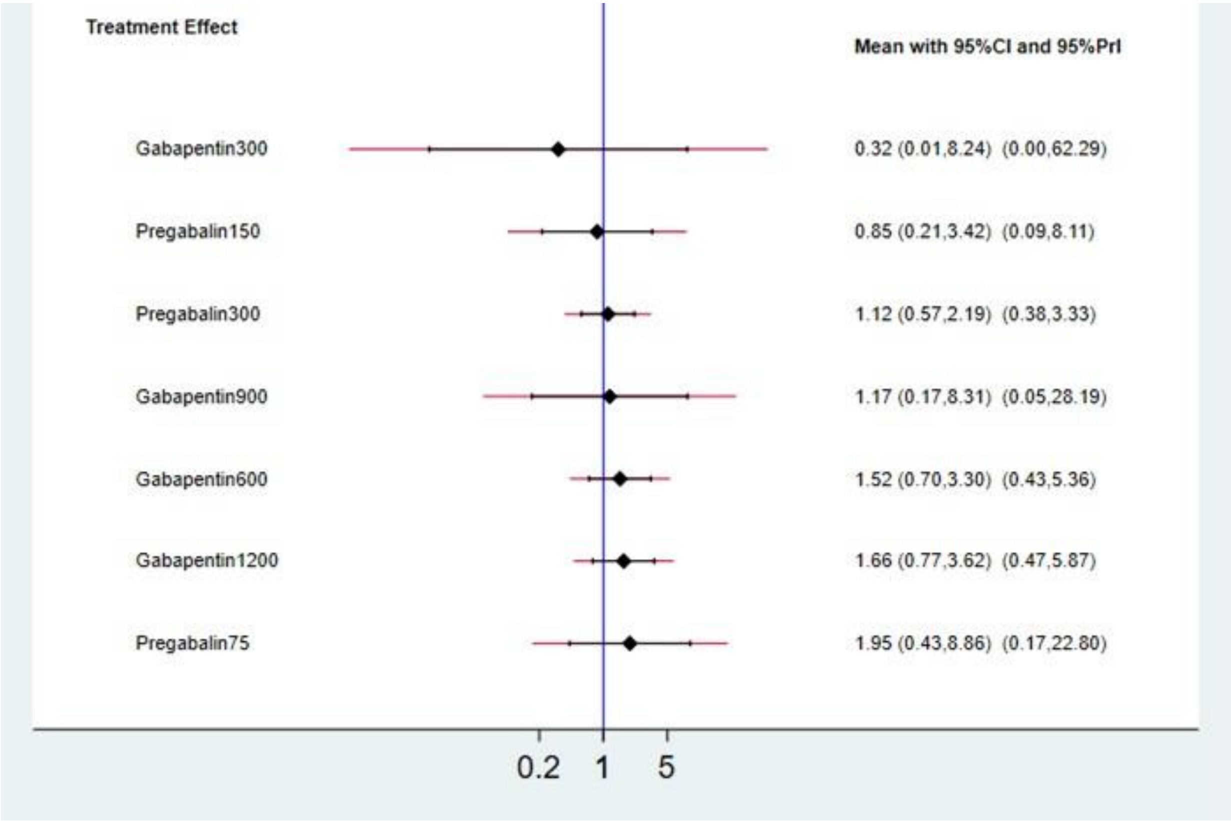

© 2023 Tsai SHL et al. *JAMA Network Open.*

eFigure 6: Forest Plot of Pairwise Comparison for Each Outcome

eFigure 6a: VAS: (A: placebo, B: Gabapentin 300mg, C: Gabapentin 400mg, D: Gabapentin 600 mg, E: Gabapentin 800mg, F: Gabapentin 900mg, G: Gabapentin 1200mg, H: pregabalin 75mg, I: pregabalin 150mg J: pregabalin 300mg)

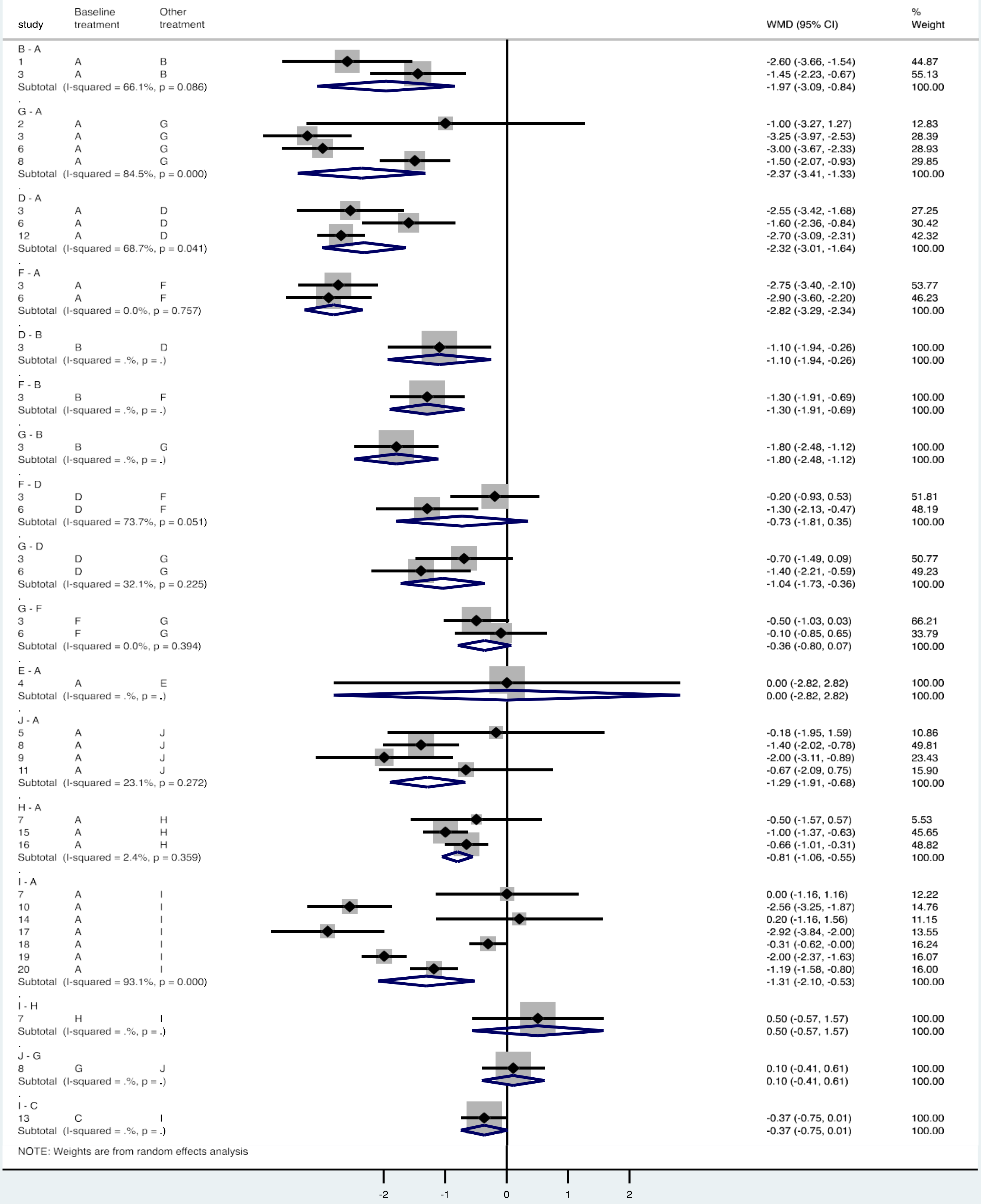

© 2023 Tsai SHL et al. *JAMA Network Open.*

eFigure 6b: Opioid consumption
(A: placebo, B: Gabapentin 300mg, C: Gabapentin 600 mg, D: Gabapentin 800mg, E: Gabapentin 900mg F: Gabapentin 1200mg, G: pregabalin 75mg, H: pregabalin 150mg, I: pregabalin 300mg)

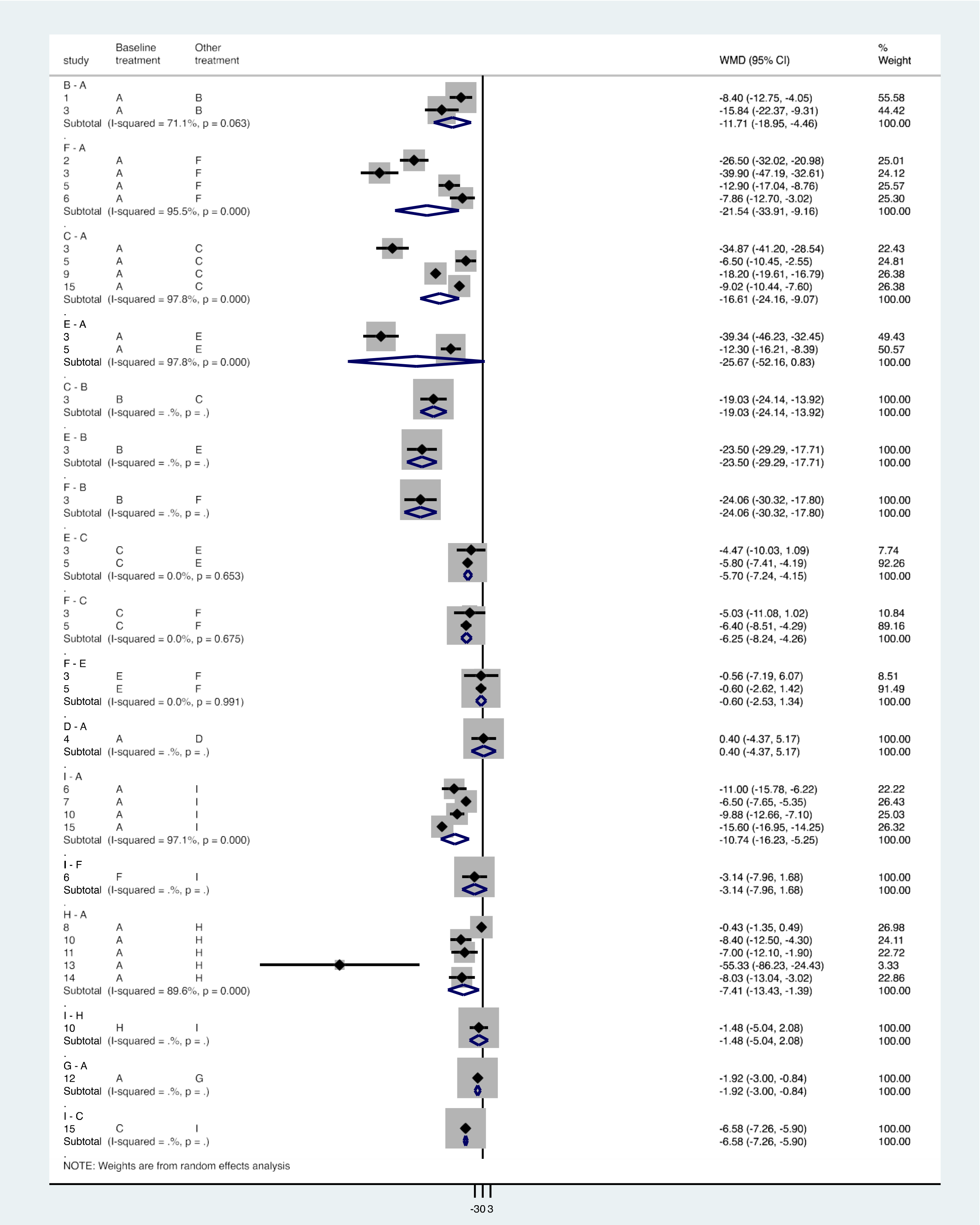

© 2023 Tsai SHL et al. *JAMA Network Open.*

eFigure 6c: Nausea

(A: placebo, B: Gabapentin 300mg, C: Gabapentin 600 mg, D: Gabapentin 800mg, E: Gabapentin 900mg, F: Gabapentin 1200mg, G: pregabalin 75mg, H: pregabalin 150mg, I: pregabalin 300mg)

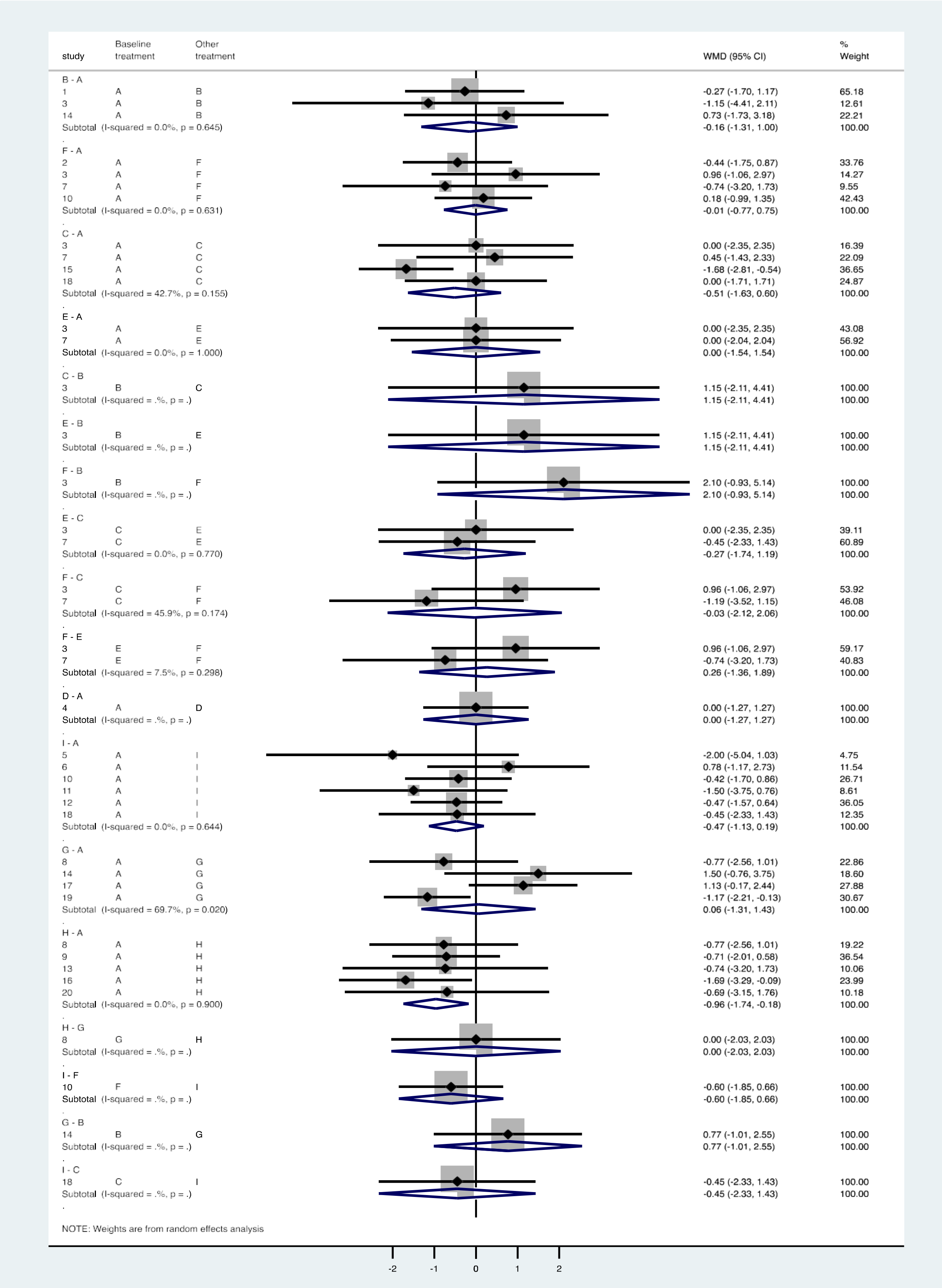

© 2023 Tsai SHL et al. *JAMA Network Open.*

eFigure 6d: Vomit

(A: placebo, B: Gabapentin 300mg, C: Gabapentin 600 mg, D: Gabapentin 800mg, E: Gabapentin 900mg, F: Gabapentin 1200mg, G: pregabalin 75mg, H: pregabalin 150mg, I: pregabalin 300mg)

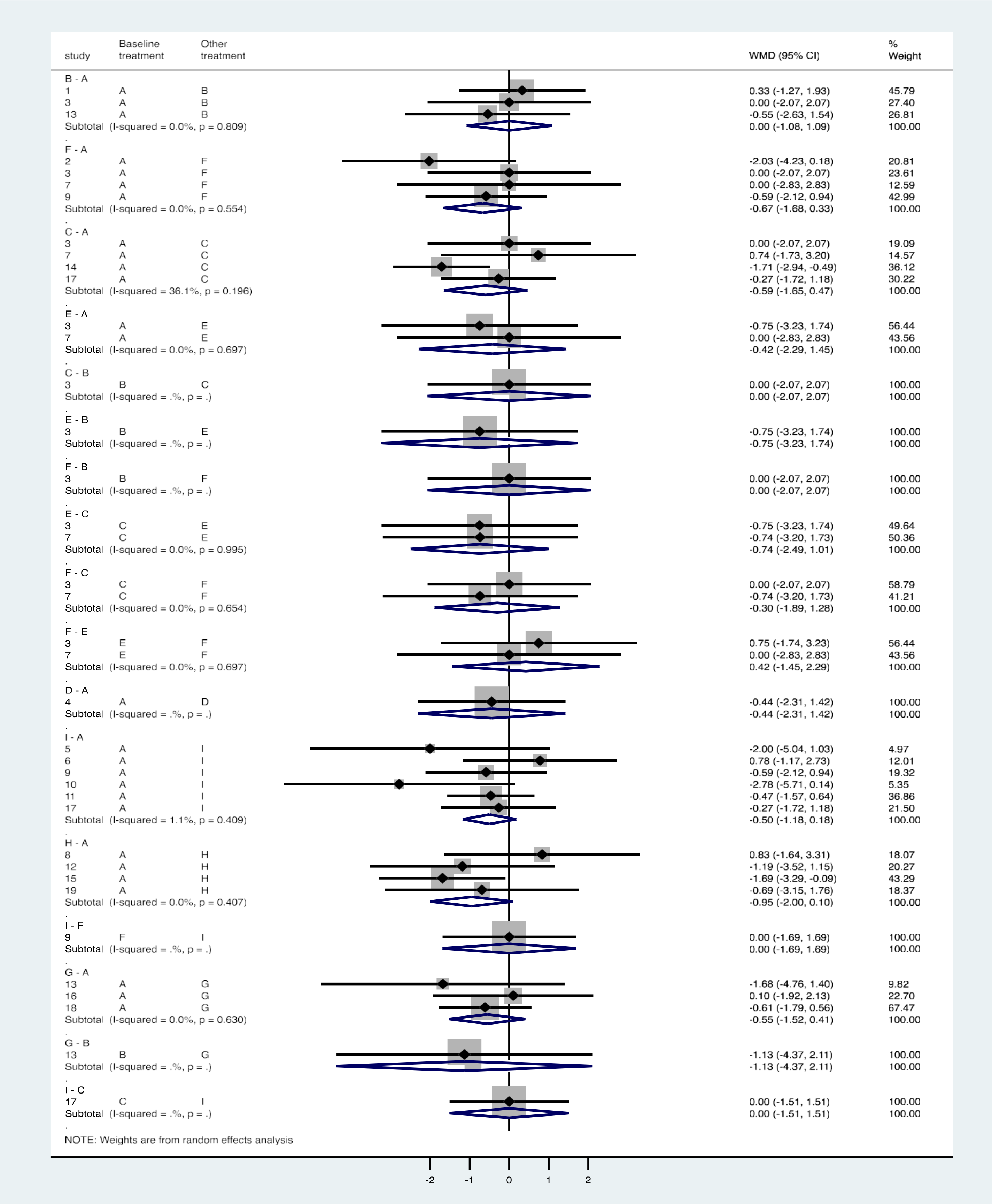

© 2023 Tsai SHL et al. *JAMA Network Open.*

eFigure 6e: Dizziness

(A: placebo, B: Gabapentin 300mg, C: Gabapentin 600 mg, D: Gabapentin 900mg, E: Gabapentin 1200mg, F: pregabalin 75mg, G: pregabalin 150mg, H: pregabalin 300mg)

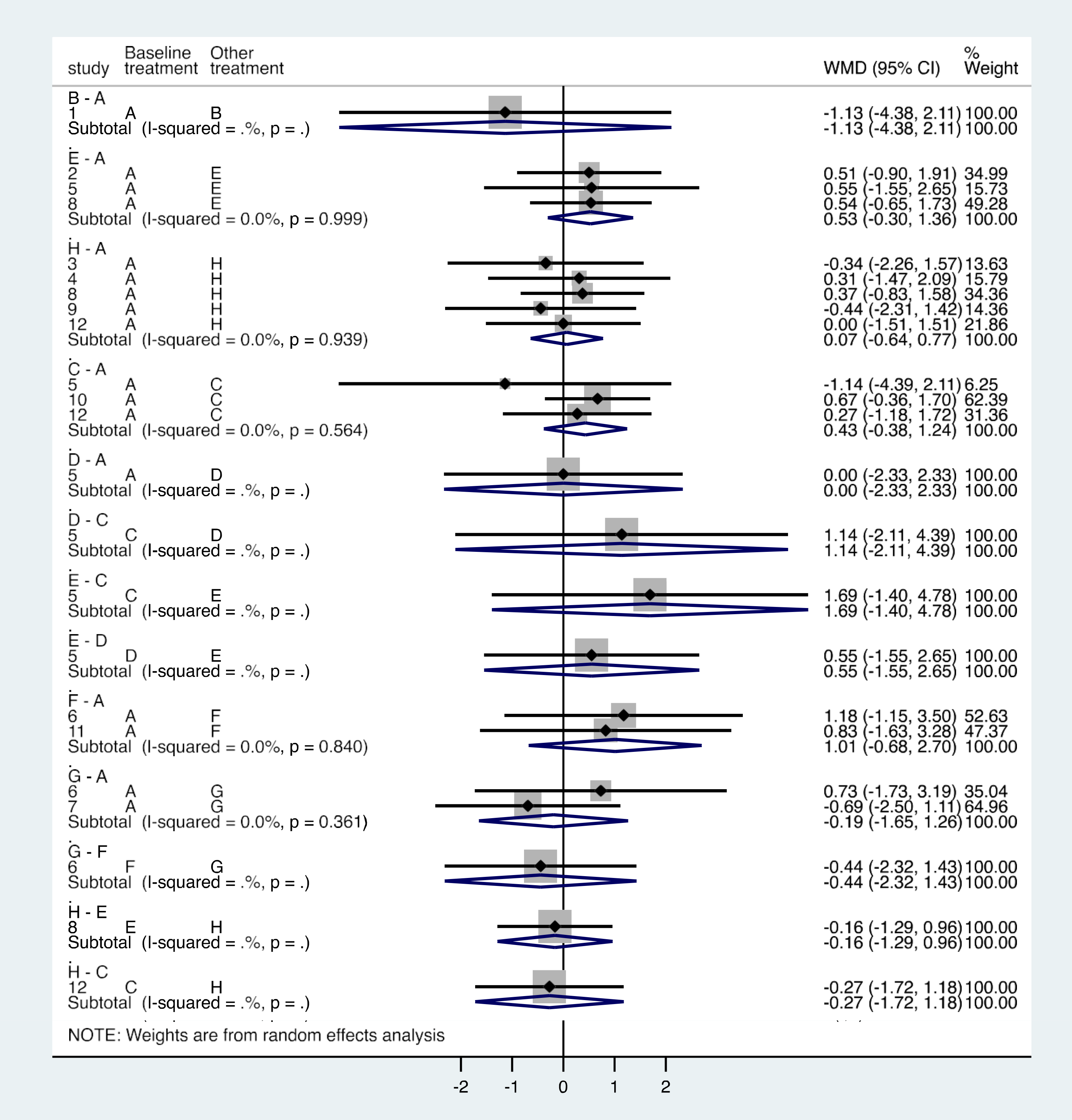

© 2023 Tsai SHL et al. *JAMA Network Open.*

eFigure 7: Publication Bias Funnel Plot

A: VAS

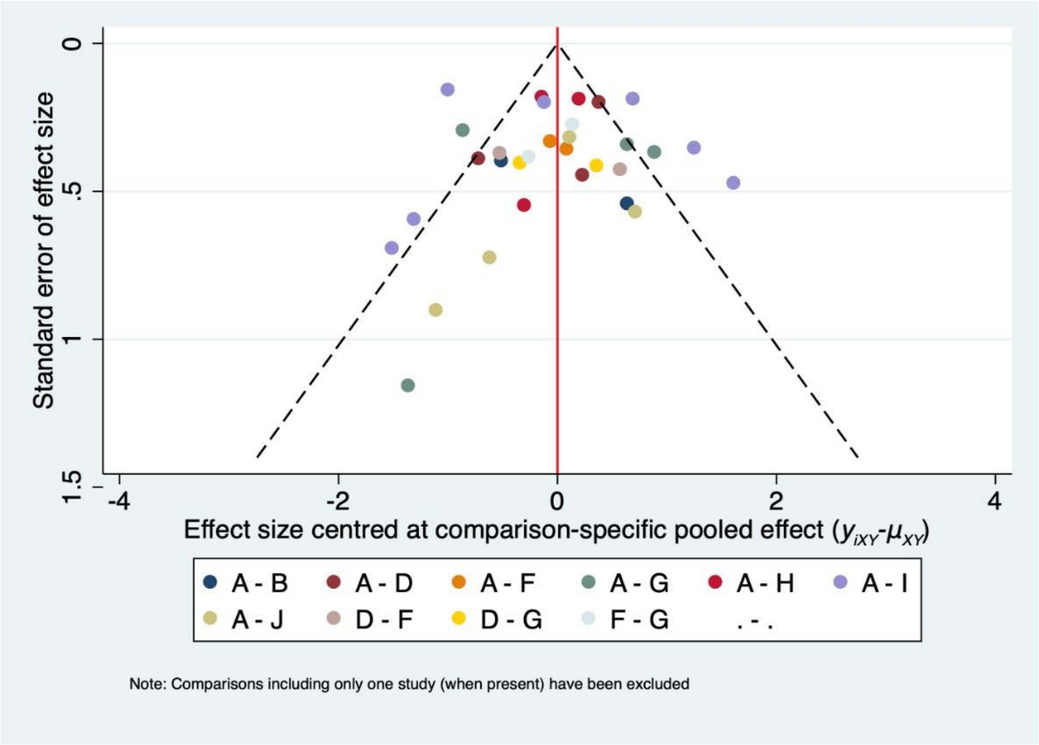

B: Opioid consumption

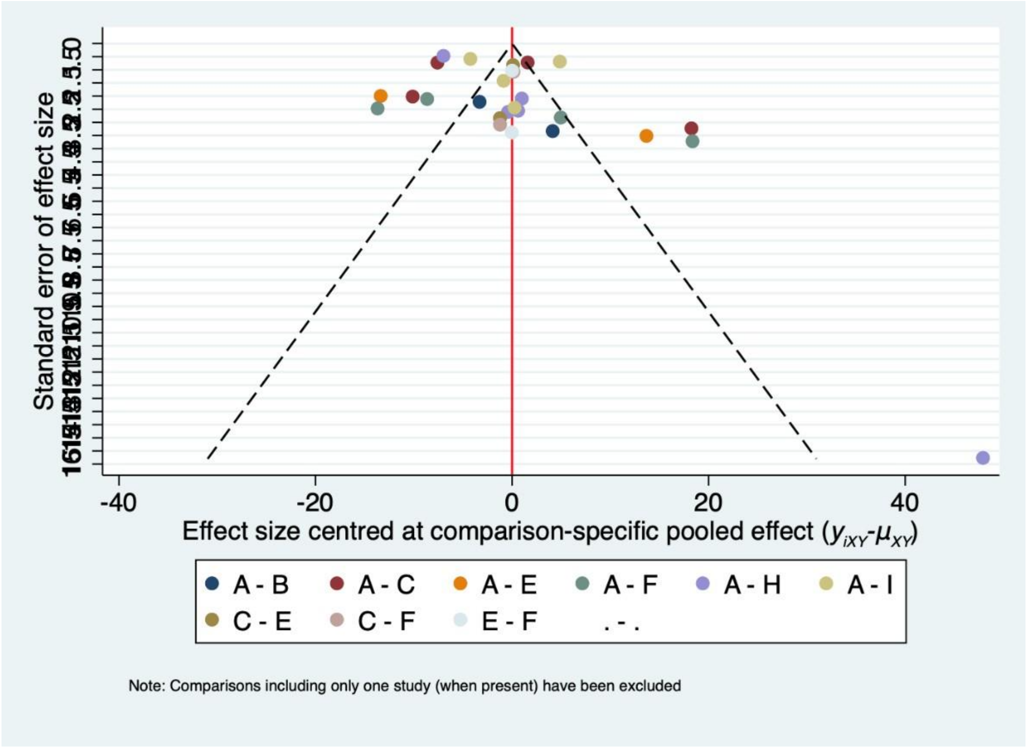

© 2023 Tsai SHL et al. *JAMA Network Open.*

C: Nausea

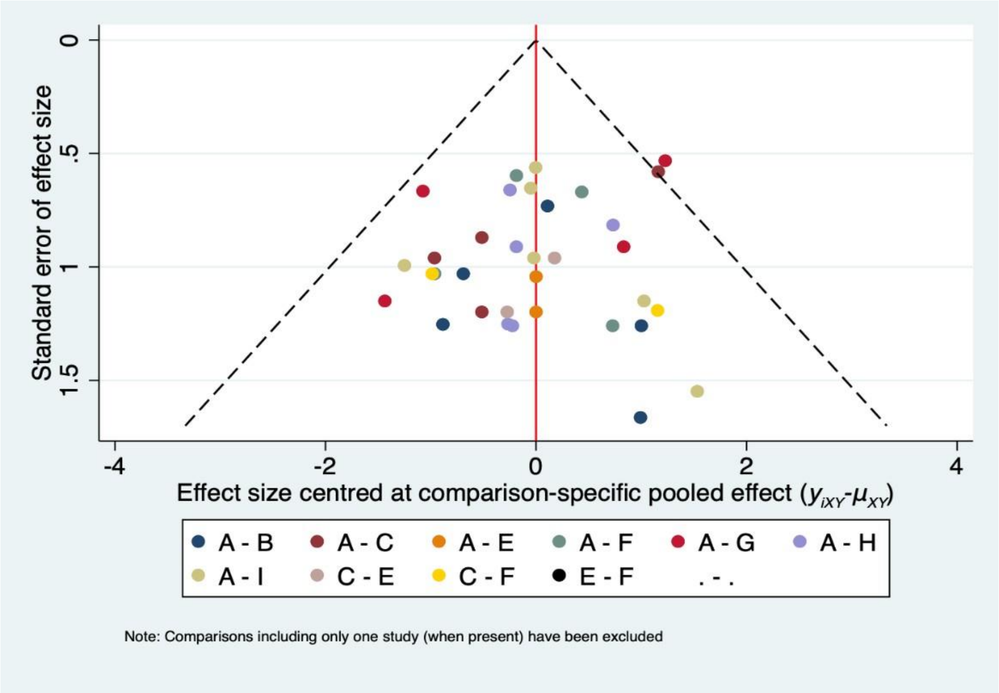

D: Vomit

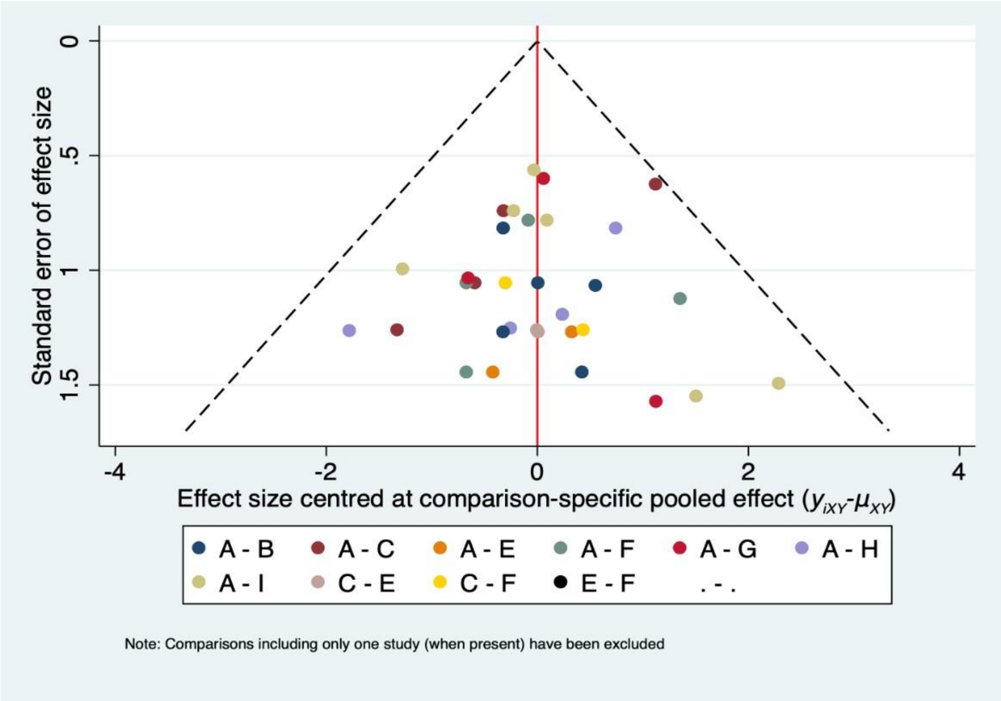

© 2023 Tsai SHL et al. *JAMA Network Open.*

E: Dizziness

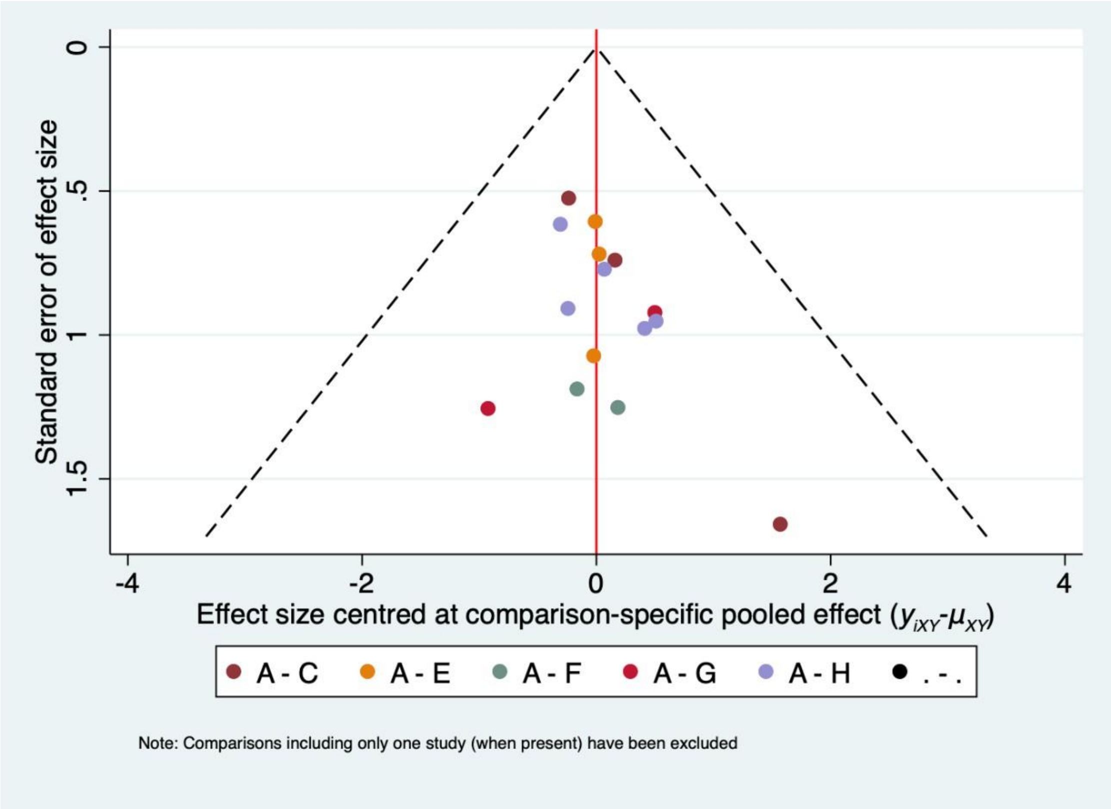

© 2023 Tsai SHL et al. *JAMA Network Open.*

eFigure 8: Publication Bias: The Egger Test

A: VAS

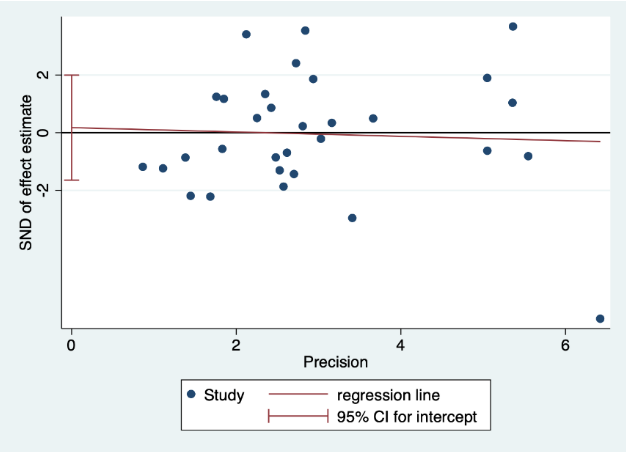

B: Opioid consumption

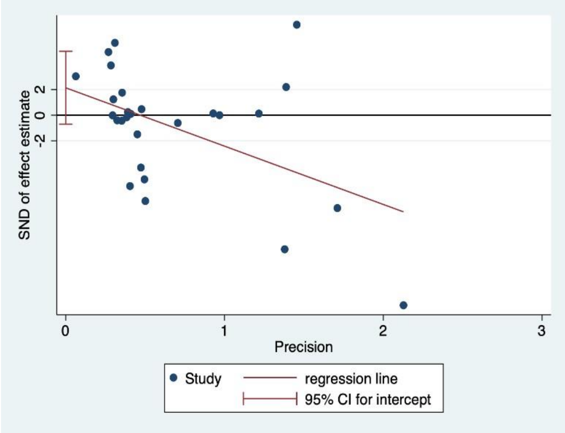

C: Nausea

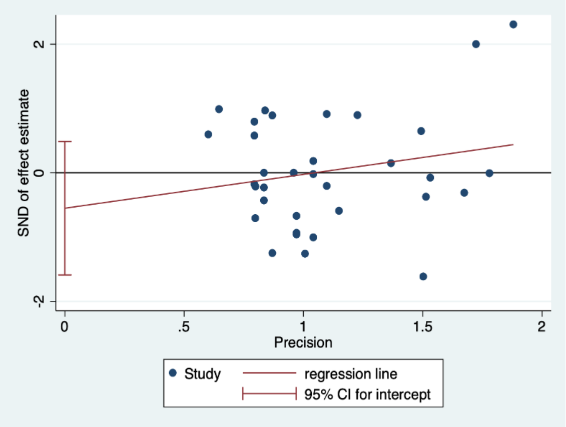

D: Vomit

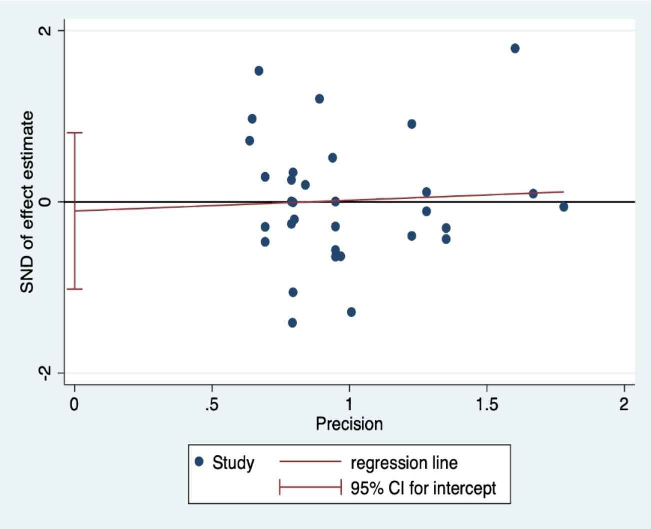

E: Dizziness

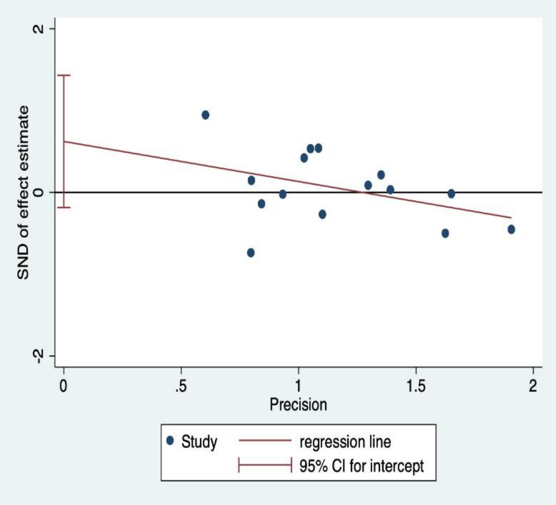

© 2023 Tsai SHL et al. *JAMA Network Open.*

eFigure 9: CINeMA for the Perioperative Outcomes (VAS, Opioid Consumption, Nausea, Vomit, Dizziness)

Study limitations (Within study bias): ROB (Cochrane risk-of-bias tool for randomized trials) was assessed and “Average Risk of Bias” was implemented in CINeMA to calculate the within study bias.

Publication bias (Across studies bias): Since p value was < 0.05 from Egger test and no obvious asymmetry was noticed in funnel plot, “all at low risk” was set in CINeMA.

Indirectness: we can assume all studies in our analysis had similar distribution in terms of their study population (spinal surgery), intervention (gabapentin/pregabalin) and outcome assessments (VAS, morphine, nausea, etc.) Thus, we judged that there was no serious concern in this domain.

Imprecision: In CINeMA setting, we considered a clinically meaningful threshold for mean difference (MD) to be 1 for VAS [1], and 10mg IV morphine for opioid consumption due to its most common IV form dosage and our analysis showing that the least significant morphine consumption is around 10 mg (Supplementary Figure 5B). Similarly, odds ratio as clinically importance was set to 0.4 for nausea, vomit and dizziness.

Heterogeneity: According to CINeMA system, heterogeneity was evaluated through comparing the clinical inference based on the 95% confidence intervals (CI) and 95% prediction interval (PI). [2]

Incoherence (Inconsistency): For inconsistency design-by-treatment interaction model was implemented. There was no significance for overall analysis with *P*>0.05 while some concerns or major concerns were noted in pair wise comparisons.

© 2023 Tsai SHL et al. *JAMA Network Open.*

eFigure 9A: CINeMA for VAS

				Mixed evidence				
Gabapentin1200 vs Gabapentin300	1	Some concerns	Low risk	No concerns	Some concerns	Some concerns	No concerns	Low
Gabapentin1200 vs Gabapentin600	2	Some concerns	Low risk	No concerns	Some concerns	Some concerns	Major concerns	Very low
Gabapentin1200 vs Gabapentin900	2	Some concerns	Low risk	No concerns	Some concerns	Some concerns	Major concerns	Very low
Gabapentin1200 vs Placebo	4	Some concerns	Low risk	No concerns	No concerns	No concerns	No concerns	Moderate
Gabapentin1200 vs Pregabalin300	1	Some concerns	Low risk	No concerns	Some concerns	Some concerns	No concerns	Low
Gabapentin300 vs Gabapentin600	1	Some concerns	Low risk	No concerns	Some concerns	Some concerns	No concerns	Low
Gabapentin300 vs Gabapentin900	1	Some concerns	Low risk	No concerns	Some concerns	Some concerns	No concerns	Low
Gabapentin300 vs Placebo	2	Some concerns	Low risk	No concerns	No concerns	Some concerns	No concerns	Low
Gabapentin400 vs Pregabalin150	1	Some concerns	Low risk	No concerns	Major concerns	No concerns	No concerns	Low
Gabapentin600 vs Gabapentin900	2	Some concerns	Low risk	No concerns	Some concerns	Some concerns	No concerns	Low
Gabapentin600 vs Placebo	3	Some concerns	Low risk	No concerns	No concerns	No concerns	No concerns	Moderate
Gabapentin800 vs Placebo	1	No concerns	Low risk	No concerns	Major concerns	No concerns	No concerns	Low
Gabapentin900 vs Placebo	2	Some concerns	Low risk	No concerns	No concerns	No concerns	No concerns	Moderate
Placebo vs Pregabalin150	7	Some concerns	Low risk	No concerns	No concerns	Some concerns	No concerns	Low
Placebo vs Pregabalin300	4	Some concerns	Low risk	No concerns	No concerns	Some concerns	No concerns	Low
Placebo vs Pregabalin75	3	No concerns	Low risk	No concerns	Some concerns	Some concerns	No concerns	Moderate
Pregabalin150 vs Pregabalin75	1	No concerns	Low risk	No concerns	Some concerns	Some concerns	No concerns	Moderate

© 2023 Tsai SHL et al. *JAMA Network Open.*

Indirect evidence								
Gabapentin1200 vs Gabapentin400	--	Some concerns	Low risk	No concerns	Some concerns	Some concerns	No concerns	Low
Gabapentin1200 vs Gabapentin800	--	Some concerns	Low risk	No concerns	Some concerns	Some concerns	No concerns	Moderate
Gabapentin1200 vs Pregabalin150	--	Some concerns	Low risk	No concerns	No concerns	Some concerns	No concerns	Low
Gabapentin1200 vs Pregabalin75	--	Some concerns	Low risk	No concerns	No concerns	Some concerns	No concerns	Low
Gabapentin300 vs Gabapentin400	--	Some concerns	Low risk	No concerns	Major concerns	No concerns	No concerns	Very low
Gabapentin300 vs Gabapentin800	--	No concerns	Low risk	No concerns	Major concerns	No concerns	No concerns	Low
Gabapentin300 vs Pregabalin150	--	Some concerns	Low risk	No concerns	Major concerns	No concerns	No concerns	Low
Gabapentin300 vs Pregabalin300	--	Some concerns	Low risk	No concerns	Major concerns	No concerns	No concerns	Very low
Gabapentin300 vs Pregabalin75	--	Some concerns	Low risk	No concerns	Some concerns	Some concerns	No concerns	Low
Gabapentin400 vs Gabapentin600	--	Some concerns	Low risk	No concerns	Some concerns	Some concerns	No concerns	Low
Gabapentin400 vs Gabapentin800	--	Some concerns	Low risk	No concerns	Major concerns	No concerns	No concerns	High
Gabapentin400 vs Gabapentin900	--	Some concerns	Low risk	No concerns	Some concerns	Some concerns	No concerns	Low
Gabapentin400 vs Placebo	--	Some concerns	Low risk	No concerns	Some concerns	Some concerns	No concerns	Low
Gabapentin400 vs Pregabalin300	--	Some concerns	Low risk	No concerns	Major concerns	No concerns	No concerns	Low
Gabapentin400 vs Pregabalin75	--	Some concerns	Low risk	No concerns	Major concerns	No concerns	No concerns	Very low
Gabapentin600 vs Gabapentin800	--	No concerns	Low risk	No concerns	Major concerns	No concerns	No concerns	Low
Gabapentin600 vs Pregabalin150	--	Some concerns	Low risk	No concerns	Some concerns	Some concerns	No concerns	Low
Gabapentin600 vs Pregabalin300	--	Some concerns	Low risk	No concerns	Some concerns	Some concerns	No concerns	Low

© 2023 Tsai SHL et al. *JAMA Network Open.*

Gabapentin600 vs Pregabalin75	--	Some concerns	Low risk	No concerns	Some concerns	Some concerns	No concerns	Low
Gabapentin800 vs Gabapentin900	--	Some concerns	Low risk	No concerns	Some concerns	Some concerns	No concerns	Low
Gabapentin800 vs Pregabalin150	--	No concerns	Low risk	No concerns	Major concerns	No concerns	No concerns	Low
Gabapentin800 vs Pregabalin300	--	No concerns	Low risk	No concerns	Major concerns	No concerns	No concerns	Low
Gabapentin800 vs Pregabalin75	--	No concerns	Low risk	No concerns	Major concerns	No concerns	No concerns	Low
Gabapentin900 vs Pregabalin150	--	Some concerns	Low risk	No concerns	No concerns	Some concerns	No concerns	Low
Gabapentin900 vs Pregabalin300	--	Some concerns	Low risk	No concerns	Some concerns	Some concerns	No concerns	Low
Gabapentin900 vs Pregabalin75	--	Some concerns	Low risk	No concerns	No concerns	Some concerns	No concerns	Low
Pregabalin150 vs Pregabalin300	--	Some concerns	Low risk	No concerns	Major concerns	No concerns	No concerns	Very low
Pregabalin300 vs Pregabalin75	--	Some concerns	Low risk	No concerns	Some concerns	Some concerns	No concerns	Low

© 2023 Tsai SHL et al. *JAMA Network Open*.

eFigure 9B CINeMA for opioid consumption

Comparison	Number of Studies	Within-study bias	Reporting bias	Indirectness	Imprecision	Heterogeneity	Incoherence	Confidence rating
Mixed evidence								
Gabapentin1200 vs Gabapentin300	1	Some concerns	Low risk	No concerns	No concerns	Some concerns	No concerns	Low
Gabapentin1200 vs Gabapentin600	2	Some concerns	Low risk	No concerns	Some concerns	Some concerns	No concerns	Low
Gabapentin1200 vs Gabapentin900	2	Some concerns	Low risk	No concerns	Some concerns	Some concerns	No concerns	Low
Gabapentin1200 vs Placebo	4	Some concerns	Low risk	No concerns	No concerns	No concerns	No concerns	Moderate
Gabapentin1200 vs Pregabalin300	1	Some concerns	Low risk	No concerns	Some concerns	Some concerns	No concerns	Low
Gabapentin300 vs Gabapentin600	1	Some concerns	Low risk	No concerns	Some concerns	No concerns	No concerns	Low
Gabapentin300 vs Gabapentin900	1	Some concerns	Low risk	No concerns	No concerns	Some concerns	No concerns	Low
Gabapentin300 vs Placebo	2	Some concerns	Low risk	No concerns	Some concerns	Some concerns	Major concerns	Very low
Gabapentin600 vs Gabapentin900	2	Some concerns	Low risk	No concerns	Some concerns	Some concerns	No concerns	Low
Gabapentin600 vs Placebo	4	Some concerns	Low risk	No concerns	No concerns	Some concerns	No concerns	Low
Gabapentin600 vs Pregabalin300	1	Some concerns	Low risk	No concerns	Some concerns	Some concerns	No concerns	Low
Gabapentin800 vs Placebo	1	No concerns	Low risk	No concerns	Major concerns	No concerns	Major concerns	Very low
Gabapentin900 vs Placebo	2	Some concerns	Low risk	No concerns	No concerns	No concerns	No concerns	Moderate
Placebo vs Pregabalin150	5	Some concerns	Low risk	No concerns	No concerns	Some concerns	No concerns	Low
Placebo vs Pregabalin300	4	Some concerns	Low risk	No concerns	No concerns	Some concerns	Some concerns	Very low
Placebo vs Pregabalin75	1	Some concerns	Low risk	No concerns	Major concerns	No concerns	Major concerns	Very low
Pregabalin150 vs Pregabalin300	1	Some concerns	Low risk	No concerns	Some concerns	Some concerns	No concerns	Low

© 2023 Tsai SHL et al. *JAMA Network Open.*

Indirect evidence								
Gabapentin1200 vs Gabapentin800	--	No concerns	Low risk	No concerns	No concerns	Some concerns	Major concerns	Very low
Gabapentin1200 vs Pregabalin150	--	Some concerns	Low risk	No concerns	No concerns	Some concerns	Major concerns	Very low
Gabapentin1200 vs Pregabalin75	--	Some concerns	Low risk	No concerns	No concerns	Some concerns	Major concerns	Very low
Gabapentin300 vs Gabapentin800	--	No concerns	Low risk	No concerns	Major concerns	No concerns	Major concerns	Very low
Gabapentin300 vs Pregabalin150	--	Some concerns	Low risk	No concerns	Some concerns	Some concerns	Major concerns	Very low
Gabapentin300 vs Pregabalin300	--	Some concerns	Low risk	No concerns	Some concerns	Some concerns	Major concerns	Very low
Gabapentin300 vs Pregabalin75	--	Some concerns	Low risk	No concerns	Major concerns	No concerns	Major concerns	Very low
Gabapentin600 vs Gabapentin800	--	No concerns	Low risk	No concerns	Some concerns	No concerns	Major concerns	Very low
Gabapentin600 vs Pregabalin150	--	Some concerns	Low risk	No concerns	Some concerns	Some concerns	Major concerns	Very low
Gabapentin600 vs Pregabalin75	--	Some concerns	Low risk	No concerns	Some concerns	Some concerns	Major concerns	Very low
Gabapentin800 vs Gabapentin900	--	Some concerns	Low risk	No concerns	No concerns	Some concerns	Major concerns	Very low
Gabapentin800 vs Pregabalin150	--	No concerns	Low risk	No concerns	Some concerns	Some concerns	Major concerns	Very low
Gabapentin800 vs Pregabalin300	--	No concerns	Low risk	No concerns	Some concerns	Some concerns	Major concerns	Very low
Gabapentin800 vs Pregabalin75	--	Some concerns	Low risk	No concerns	Major concerns	No concerns	Major concerns	Very low
Gabapentin900 vs Pregabalin150	--	Some concerns	Low risk	No concerns	No concerns	Some concerns	Major concerns	Very low
Gabapentin900 vs Pregabalin300	--	Some concerns	Low risk	No concerns	Some concerns	Some concerns	Major concerns	Very low
Gabapentin900 vs Pregabalin75	--	Some concerns	Low risk	No concerns	No concerns	Some concerns	Major concerns	Very low
Pregabalin150 vs Pregabalin75	--	Some concerns	Low risk	No concerns	Some concerns	Some concerns	Major concerns	Very low
Pregabalin300 vs Pregabalin75	--	Some concerns	Low risk	No concerns	Some concerns	Some concerns	Major concerns	Very low

© 2023 Tsai SHL et al. *JAMA Network Open.*

eFigure 9C: CINeMA for Nausea

Comparison	Number of Studies	Within-study bias	Reporting bias	Indirectness	Imprecision	Heterogeneity	Incoherence	Confidence rating
Mixed evidence								
Gabapentin1200 vs Gabapentin300	1	Some concerns	Low risk	No concerns	Some concerns	Some concerns	No concerns	Low
Gabapentin1200 vs Gabapentin600	2	Some concerns	Low risk	No concerns	Some concerns	No concerns	No concerns	Low
Gabapentin1200 vs Gabapentin900	2	Some concerns	Low risk	No concerns	Major concerns	No concerns	No concerns	Very low
Gabapentin1200 vs Placebo	4	Some concerns	Low risk	No concerns	No concerns	Major concerns	No concerns	Very low
Gabapentin1200 vs Pregabalin300	1	Some concerns	Low risk	No concerns	Some concerns	No concerns	No concerns	Low
Gabapentin300 vs Gabapentin600	1	Some concerns	Low risk	No concerns	Major concerns	No concerns	No concerns	Very low
Gabapentin300 vs Gabapentin900	1	Some concerns	Low risk	No concerns	Major concerns	No concerns	No concerns	Very low
Gabapentin300 vs Placebo	3	No concerns	Low risk	No concerns	Some concerns	No concerns	No concerns	Moderate
Gabapentin300 vs Pregabalin75	1	Some concerns	Low risk	No concerns	Some concerns	Some concerns	No concerns	Low
Gabapentin600 vs Gabapentin900	2	Some concerns	Low risk	No concerns	Major concerns	No concerns	No concerns	Very low
Gabapentin600 vs Placebo	4	Some concerns	Low risk	No concerns	Some concerns	No concerns	No concerns	Low
Gabapentin600 vs Pregabalin300	1	Some concerns	Low risk	No concerns	Major concerns	No concerns	No concerns	Very low
Gabapentin800 vs Placebo	1	No concerns	Low risk	No concerns	Major concerns	No concerns	No concerns	Very low
Gabapentin900 vs Placebo	2	Some concerns	Low risk	No concerns	Some concerns	Some concerns	No concerns	Low
Placebo vs Pregabalin150	5	Some concerns	Low risk	No concerns	No concerns	Some concerns	No concerns	Low
Placebo vs Pregabalin300	6	No concerns	Low risk	No concerns	Some concerns	No concerns	No concerns	Low
Placebo vs Pregabalin75	4	Some concerns	Low risk	No concerns	No concerns	Some concerns	No concerns	Low
Pregabalin150 vs Pregabalin75	1	Some concerns	Low risk	No concerns	Some concerns	No concerns	No concerns	Low

© 2023 Tsai SHL et al. *JAMA Network Open.*

Indirect evidence								
Gabapentin1200 vs Gabapentin800	--	No concerns	Low risk	No concerns	Major concerns	No concerns	No concerns	Low
Gabapentin1200 vs Pregabalin150	--	Some concerns	Low risk	No concerns	Some concerns	No concerns	No concerns	Low
Gabapentin1200 vs Pregabalin75	--	Some concerns	Low risk	No concerns	Major concerns	No concerns	No concerns	Very low
Gabapentin300 vs Gabapentin800	--	No concerns	Low risk	No concerns	Major concerns	No concerns	No concerns	Low
Gabapentin300 vs Pregabalin150	--	Some concerns	Low risk	No concerns	Major concerns	No concerns	No concerns	Very low
Gabapentin300 vs Pregabalin300	--	Some concerns	Low risk	No concerns	Major concerns	No concerns	No concerns	Very low
Gabapentin600 vs Gabapentin800	--	No concerns	Low risk	No concerns	Major concerns	No concerns	No concerns	Low
Gabapentin600 vs Pregabalin150	--	Some concerns	Low risk	No concerns	Some concerns	Some concerns	No concerns	Low
Gabapentin600 vs Pregabalin75	--	Some concerns	Low risk	No concerns	Some concerns	No concerns	No concerns	Low
Gabapentin800 vs Gabapentin900	--	Some concerns	Low risk	No concerns	Major concerns	No concerns	No concerns	Very low
Gabapentin800 vs Pregabalin150	--	No concerns	Low risk	No concerns	Some concerns	No concerns	No concerns	Moderate
Gabapentin800 vs Pregabalin300	--	No concerns	Low risk	No concerns	Major concerns	No concerns	No concerns	Low
Gabapentin800 vs Pregabalin75	--	No concerns	Low risk	No concerns	Major concerns	No concerns	No concerns	Low
Gabapentin900 vs Pregabalin150	--	Some concerns	Low risk	No concerns	Major concerns	No concerns	No concerns	Very low
Gabapentin900 vs Pregabalin300	--	Some concerns	Low risk	No concerns	Major concerns	No concerns	No concerns	Very low
Gabapentin900 vs Pregabalin75	--	Some concerns	Low risk	No concerns	Major concerns	No concerns	No concerns	Low
Pregabalin150 vs Pregabalin300	--	Some concerns	Low risk	No concerns	Some concerns	Some concerns	No concerns	Low
Pregabalin300 vs Pregabalin75	--	Some concerns	Low risk	No concerns	Some concerns	No concerns	No concerns	Low

© 2023 Tsai SHL et al. *JAMA Network Open.*

eFigure 9D: CINeMA for Vomit

Comparison	Number of Studies	Within-study bias	Reporting bias	Indirectness	Imprecision	Heterogeneity	Incoherence	Confidence rating
Mixed evidence								
Gabapentin1200 vs Gabapentin300	1	Some concerns	Low risk	No concerns	Some concerns	No concerns	No concerns	Low
Gabapentin1200 vs Gabapentin600	2	Some concerns	Low risk	No concerns	Major concerns	No concerns	No concerns	Very low
Gabapentin1200 vs Gabapentin900	2	Some concerns	Low risk	No concerns	Major concerns	No concerns	No concerns	Very low
Gabapentin1200 vs Placebo	4	Some concerns	Low risk	No concerns	Some concerns	No concerns	No concerns	Low
Gabapentin1200 vs Pregabalin300	1	Some concerns	Low risk	No concerns	Some concerns	No concerns	No concerns	Low
Gabapentin300 vs Gabapentin600	1	Some concerns	Low risk	No concerns	Some concerns	No concerns	No concerns	Low
Gabapentin300 vs Gabapentin900	1	Some concerns	Low risk	No concerns	Some concerns	Some concerns	No concerns	Low
Gabapentin300 vs Placebo	3	Some concerns	Low risk	No concerns	Some concerns	Some concerns	No concerns	Low
Gabapentin300 vs Pregabalin75	1	Some concerns	Low risk	No concerns	Major concerns	No concerns	No concerns	Very low
Gabapentin600 vs Gabapentin900	2	Some concerns	Low risk	No concerns	Major concerns	No concerns	No concerns	Very low
Gabapentin600 vs Placebo	4	Some concerns	Low risk	No concerns	No concerns	Some concerns	No concerns	Low
Gabapentin600 vs Pregabalin300	1	Some concerns	Low risk	No concerns	Some concerns	No concerns	No concerns	Low
Gabapentin800 vs Placebo	1	No concerns	Low risk	No concerns	Major concerns	No concerns	No concerns	Low
Gabapentin900 vs Placebo	2	Some concerns	Low risk	No concerns	Some concerns	No concerns	No concerns	Low
Placebo vs Pregabalin150	4	Some concerns	Low risk	No concerns	Some concerns	No concerns	No concerns	Low
Placebo vs Pregabalin300	6	No concerns	Low risk	No concerns	Some concerns	No concerns	No concerns	Moderate
Placebo vs Pregabalin75	3	Some concerns	Low risk	No concerns	Some concerns	No concerns	No concerns	Low

© 2023 Tsai SHL et al. *JAMA Network Open.*

Indirect evidence

Gabapentin1200 vs Gabapentin800	--	No concerns	Low risk	No concerns	Major concerns	No concerns	No concerns	Low
Gabapentin1200 vs Pregabalin150	--	Some concerns	Low risk	No concerns	Major concerns	No concerns	No concerns	Low
Gabapentin1200 vs Pregabalin75	--	Some concerns	Low risk	No concerns	Major concerns	No concerns	No concerns	Very low
Gabapentin300 vs Gabapentin800	--	No concerns	Low risk	No concerns	Major concerns	No concerns	No concerns	Low
Gabapentin300 vs Pregabalin150	--	Some concerns	Low risk	No concerns	Some concerns	No concerns	No concerns	Low
Gabapentin300 vs Pregabalin300	--	Some concerns	Low risk	No concerns	Some concerns	No concerns	No concerns	Low
Gabapentin600 vs Gabapentin800	--	No concerns	Low risk	No concerns	Major concerns	No concerns	No concerns	Low
Gabapentin600 vs Pregabalin150	--	Some concerns	Low risk	No concerns	Major concerns	No concerns	No concerns	Very low
Gabapentin600 vs Pregabalin75	--	Some concerns	Low risk	No concerns	Major concerns	No concerns	No concerns	Very low
Gabapentin800 vs Gabapentin900	--	Some concerns	Low risk	No concerns	Major concerns	No concerns	No concerns	Very low
Gabapentin800 vs Pregabalin150	--	No concerns	Low risk	No concerns	Major concerns	No concerns	No concerns	Low
Gabapentin800 vs Pregabalin300	--	No concerns	Low risk	No concerns	Major concerns	No concerns	No concerns	Low
Gabapentin800 vs Pregabalin75	--	No concerns	Low risk	No concerns	Major concerns	No concerns	No concerns	Low
Gabapentin900 vs Pregabalin150	--	Some concerns	Low risk	No concerns	Major concerns	No concerns	No concerns	Very low
Gabapentin900 vs Pregabalin300	--	Some concerns	Low risk	No concerns	Major concerns	No concerns	No concerns	Very low
Gabapentin900 vs Pregabalin75	--	Some concerns	Low risk	No concerns	Major concerns	No concerns	No concerns	Very low
Pregabalin150 vs Pregabalin300	--	Some concerns	Low risk	No concerns	Some concerns	Some concerns	No concerns	Low
Pregabalin150 vs Pregabalin75	--	Some concerns	Low risk	No concerns	Major concerns	No concerns	No concerns	Very low
Pregabalin300 vs Pregabalin75	--	Some concerns	Low risk	No concerns	Major concerns	No concerns	No concerns	Very low

© 2023 Tsai SHL et al. *JAMA Network Open.*

eFigure 9E CINeMA for Dizziness

Comparison	Number of Studies	Within-study bias	Reporting bias	Indirectness	Imprecision	Heterogeneity	Incoherence	Confidence rating
Mixed evidence								
Gabapentin1200 vs Gabapentin600	1	Some concerns	Low risk	No concerns	Major concerns	No concerns	No concerns	Very low
Gabapentin1200 vs Gabapentin900	1	Some concerns	Low risk	No concerns	Major concerns	No concerns	No concerns	Very low
Gabapentin1200 vs Placebo	3	Some concerns	Low risk	No concerns	Some concerns	No concerns	No concerns	Low
Gabapentin1200 vs Pregabalin300	1	Some concerns	Low risk	No concerns	Some concerns	No concerns	No concerns	Low
Gabapentin300 vs Placebo	1	No concerns	Low risk	No concerns	Major concerns	No concerns	No concerns	Low
Gabapentin600 vs Gabapentin900	1	Some concerns	Low risk	No concerns	Major concerns	No concerns	No concerns	Very low
Gabapentin600 vs Placebo	3	No concerns	Low risk	No concerns	Some concerns	No concerns	No concerns	Moderate
Gabapentin600 vs Pregabalin300	1	Some concerns	Low risk	No concerns	Some concerns	No concerns	No concerns	Low
Gabapentin900 vs Placebo	1	Some concerns	Low risk	No concerns	Major concerns	No concerns	No concerns	Very low
Placebo vs Pregabalin150	2	Some concerns	Low risk	No concerns	Major concerns	No concerns	No concerns	Very low
Placebo vs Pregabalin300	5	Some concerns	Low risk	No concerns	No concerns	No concerns	No concerns	Moderate
Placebo vs Pregabalin75	2	No concerns	Low risk	No concerns	Some concerns	Some concerns	No concerns	Moderate
Pregabalin150 vs Pregabalin75	1	No concerns	Low risk	No concerns	Some concerns	Some concerns	No concerns	Moderate

© 2023 Tsai SHL et al. *JAMA Network Open.*

Indirect evidence								
Gabapentin1200 vs Gabapentin300	--	No concerns	Low risk	No concerns	Major concerns	No concerns	No concerns	Very low
Gabapentin1200 vs Pregabalin150	--	Some concerns	Low risk	No concerns	Major concerns	No concerns	No concerns	Very low
Gabapentin1200 vs Pregabalin75	--	No concerns	Low risk	No concerns	Major concerns	No concerns	No concerns	Low
Gabapentin300 vs Gabapentin600	--	No concerns	Low risk	No concerns	Major concerns	No concerns	No concerns	Low
Gabapentin300 vs Gabapentin900	--	Some concerns	Low risk	No concerns	Major concerns	No concerns	No concerns	Very low
Gabapentin300 vs Pregabalin150	--	No concerns	Low risk	No concerns	Major concerns	No concerns	No concerns	Low
Gabapentin300 vs Pregabalin300	--	No concerns	Low risk	No concerns	Major concerns	No concerns	No concerns	Low
Gabapentin300 vs Pregabalin75	--	No concerns	Low risk	No concerns	Major concerns	No concerns	No concerns	Low
Gabapentin600 vs Pregabalin150	--	Some concerns	Low risk	No concerns	Major concerns	No concerns	No concerns	Very low
Gabapentin600 vs Pregabalin75	--	No concerns	Low risk	No concerns	Major concerns	No concerns	No concerns	Low
Gabapentin900 vs Pregabalin150	--	Some concerns	Low risk	No concerns	Major concerns	No concerns	No concerns	Very low
Gabapentin900 vs Pregabalin300	--	Some concerns	Low risk	No concerns	Major concerns	No concerns	No concerns	Very low
Gabapentin900 vs Pregabalin75	--	Some concerns	Low risk	No concerns	Major concerns	No concerns	No concerns	Very low
Pregabalin150 vs Pregabalin300	--	Some concerns	Low risk	No concerns	Major concerns	No concerns	No concerns	Very low
Pregabalin300 vs Pregabalin75	--	No concerns	Low risk	No concerns	Major concerns	No concerns	No concerns	Low

© 2023 Tsai SHL et al. *JAMA Network Open.*

eTable 1. Electronic Database Search Strategy

OVID

Database(s): **Ovid MEDLINE(R) 1946 to Present and Epub Ahead of Print, In-Process & Other Non-Indexed Citations and Ovid MEDLINE(R) Daily, EBM Reviews - Cochrane Central Register of Controlled Trials** July 2021**, EBM Reviews - Cochrane Database of Systematic Reviews** 2005 to August 25, 2021**, Embase** 1974 to 2021 August 27
Search Strategy:

#	Searches
1	exp Spinal Fusion/
2	exp Spine/su
3	((spine or spinal or lumbar or lumbosacral or cervico* or cervical or thoracic or thoracolumbar or occipitocervical or "occipito-cervical" or occipitalcervical or "occipital-cervical" or atlantoaxial or "atlanto-axial" or occipitoatlantoaxial or "occipoto-atlantoaxial" or "craniovertebral junction" or subluxation or vertebr* or intervertebral or disc or discs or disk or disks or sacral or sacrum or "trans-sacr*") adj10 (fusion* or fused or fusing or fixation or decompression or surg* or operat* or arthrodesis or reconstruct*)).ti,ab,hw,kw.
4	(discectom* or diskectom* or laminectomy or spondylosyndesis).ti,ab,hw,kw.
5	exp Decompression, Surgical/
6	spinal cord decompression/
7	exp Diskectomy/
8	Pedicle Screws/ or screw*.ti.
9	or/1-8
10	Pregabalin/
11	(pregabalin or lyrica).mp.
12	10 or 11
13	exp Pain, Postoperative/
14	pain.ti. or (pain adj7 (after* or post* or surg* or operat*)).ti,ab.
15	13 or 14
16	9 and 12 and 15
17	(conference abstract or conference review or editorial or erratum or note or addresses or autobiography or bibliography or biography or blogs or comment or dictionary or directory or interactive tutorial or interview or lectures or legal cases or legislation or news or newspaper article or patient education handout or periodical index or portraits or published erratum or video-audio media or webcasts).mp. or conference abstract.st.
18	16 not 17
19	(exp animals/ or exp nonhuman/) not exp humans/
20	((alpaca or alpacas or amphibian or amphibians or animal or animals or antelope or armadillo or armadillos or avian or baboon or baboons or beagle or beagles or bee or bees or bird or birds or bison or bovine or buffalo or buffaloes or buffalos or "c elegans" or "Caenorhabditis elegans" or camel or camels or canine or canines or carp or cats or cattle or chick or chicken or chickens or chicks or chimp or chimpanze or chimpanzees

© 2023 Tsai SHL et al. *JAMA Network Open.*

	or chimps or cow or cows or "D melanogaster" or "dairy calf" or "dairy calves" or deer or dog or dogs or donkey or donkeys or drosophila or "Drosophila melanogaster" or duck or duckling or ducklings or ducks or equid or equids or equine or equines or feline or felines or ferret or ferrets or finch or finches or fish or flatworm or flatworms or fox or foxes or frog or frogs or "fruit flies" or "fruit fly" or "G mellonella" or "Galleria mellonella" or geese or gerbil or gerbils or goat or goats or goose or gorilla or gorillas or hamster or hamsters or hare or hares or heifer or heifers or horse or horses or insect or insects or jellyfish or kangaroo or kangaroos or kitten or kittens or lagomorph or lagomorphs or lamb or lambs or llama or llamas or macaque or macaques or macaw or macaws or marmoset or marmosets or mice or minipig or minipigs or mink or minks or monkey or monkeys or mouse or mule or mules or nematode or nematodes or octopus or octopuses or orangutan or "orang-utan" or orangutans or "orang-utans" or oxen or parrot or parrots or pig or pigeon or pigeons or piglet or piglets or pigs or porcine or primate or primates or quail or rabbit or rabbits or rat or rats or reptile or reptiles or rodent or rodents or ruminant or ruminants or salmon or sheep or shrimp or slug or slugs or swine or tamarin or tamarins or toad or toads or trout or urchin or urchins or vole or voles or waxworm or waxworms or worm or worms or xenopus or "zebra fish" or zebrafish) not (human or humans or patient or patients)).ti,ab,hw,kw.
21	(rat or rats or mice or mouse or murine or pig or pigs or porcine or swine or dog or dogs).ti.
22	or/19-21
23	18 not 22
24	remove duplicates from 23

© 2023 Tsai SHL et al. *JAMA Network Open.*

SCOPUS

1	TITLE-ABS-KEY ((spine OR spinal OR lumbar OR lumbosacral OR cervico* OR cervical OR thoracic OR thoracolumbar OR occipitocervical OR "occipito-cervical" OR occipitalcervical OR "occipital-cervical" OR atlantoaxial OR "atlanto-axial" OR occipitoatlantoaxial OR "occipoto-atlantoaxial" OR "craniovertebral junction" OR subluxation OR vertebr* OR intervertebral OR disc OR discs OR disk OR disks OR sacral OR sacrum OR "trans-sacr*") W/8 (fusion* OR fused OR fusing OR fixation OR decompression OR surg* OR operat* OR arthrodesis OR reconstruct*))
2	TITLE-ABS-KEY (pregabalin or lyrica)
3	TITLE (pain) or TITLE-ABS-KEY (pain W/7 (after* or post* or surg* or operat*))
4	1 and 2 and 3
5	4 AND (LIMIT-TO (DOCTYPE,"ar")) AND (LIMIT-TO (LANGUAGE,"English"))
6	5 not INDEX(embase) OR INDEX(medline) OR PMID(0* OR 1* OR 2* OR 3* OR 4* OR 5* OR 6* OR 7* OR 8* OR 9*)

© 2023 Tsai SHL et al. *JAMA Network Open.*

eTable 2 Excluded Studies With Reasons

Author	Study Name	Reasons for exclusion
Reuben 2006	The analgesic efficacy of celecoxib, pregabalin, and their combination for spinal fusion surgery	Retracted article
ElShal 2010	Pregabalin versus ketorolac for postoperative analgesia after lumbar laminectomy (a comparative randomized controlled study)	Only abstract available
Yu 2013	Gabapentin and pregabalin in the management of postoperative pain after lumbar spinal surgery: a systematic review and meta-analysis	Wrong study design
Garcia 2013	A multimodal approach for postoperative pain management after lumbar decompression surgery: a prospective, randomized study	Wrong intervention
Dolgun 2014	Gabapentin versus pregabalin in relieving early post-surgical neuropathic pain in patients after lumbar disc herniation surgery: a prospective clinical trial	No accessible data
Lam 2015	Efficacy of pregabalin in acute postoperative pain under different surgical categories a meta-analysis	Wrong study design
Feng 2016	Preoperative single dose of pregabalin alleviates postoperative pain: Systematic review and meta-analysis	Wrong study design
Ko 2016	The effectiveness of oral corticosteroids for management of lumbar radiating pain: randomized, controlled trial study	Wrong population
Canos 2016	Preventive Analgesia with Pregabalin in Neuropathic Pain from "Failed Back Surgery Syndrome": Assessment of Sleep Quality and Disability	No accessible data
Hirai 2016	Pregabalin versus acetaminophen for a treatment of chronic neuropathic pain on extremities after cervical surgery: a prospective randomized, open-label preliminary study	No accessible data
Kim 2016	Preemptive multimodal analgesia for postoperative pain management after lumbar fusion surgery: a randomized controlled trial	Wrong population
Fujita 2016	A randomized placebo-controlled study of preoperative pregabalin for postoperative analgesia in patients with spinal surgery	No accessible data
Liu 2017	A meta-analysis of the preoperative use of gabapentinoids for the treatment of acute postoperative pain following spinal surgery	Wrong study design
Jiang 2017	Preoperative use of pregabalin for acute pain in spine surgery: A meta-analysis of randomized controlled trials	Wrong study design
Pinar 2017	Effects of Addition of Preoperative Intravenous Ibuprofen to Pregabalin on Postoperative Pain in Posterior Lumbar Interbody Fusion Surgery	Wrong intervention
Helenius 2018	Preoperative pregabalin has no effect on intraoperative neurophysiological monitoring in adolescents undergoing posterior spinal fusion for spinal deformities: a double-blind, randomized, placebo-controlled clinical trial	No accessible data
Omara 2019	The Effect Of The Use Of Pre-Emptive Oral Pregabalin On The Postoperative Spinal Analgesia In Patients Presented For Orthopedic Surgeries: Randomized Controlled Trial	Wrong population

© 2023 Tsai SHL et al. *JAMA Network Open.*

McEntarfer 2019	Multimodal Analgesia for Spinal Surgery - What Is the Gold Standard?	Wrong study design
Trzcinski 2019	Use of Gabapentin in Posterior Spinal Fusion is Associated with Decreased Postoperative Pain and Opioid Use in Children and Adolescents	No accessible data
Helenius 2020	Preemptive Pregabalin in Children and Adolescents Undergoing Posterior Instrumented Spinal Fusion: A Double-Blinded, Placebo-Controlled, Randomized Clinical Trial	No accessible data
Ntalouka 2021	Multimodal Analgesia in Spine Surgery: An Umbrella Review	Wrong study design
Helenius 2021	Pregabalin and Persistent Postoperative Pain Following Posterior Spinal Fusion in Children and Adolescents: A Randomized Clinical Trial	No accessible data
Bilgin 2021	Post-operative pain management for single-level lumbar disc herniation surgery: A comparison of betamethasone, ibuprofen, and pregabalin	No accessible data
NCT01168531 2010	The Effect of Pregabalin and Dexamethasone on Acute and Chronic Pain After Lumbar Spinal Surgery	No results posted
NCT02120703 2014	Pregabalin Compared to Gabapentin for Pain Control in Lumbar Disc Surgery	No results posted
TCTR20160729 003	The effect of preopertive pregabalin administration in postoperative pain control after laminectomy and instrumented fusion in degenerative lumbar spine disease: a randomized double-blind placebo controlled study	Only abstract
UMIN000028 475	The efficacy of duloxetine for neuropathic pain a comparison of pregabalin	No results posted
IRCT2017030 132832N1	Evaluation the effect of two different doses of pregabalin before surgery on postoperative pain after lumbar disc surgery	No results posted
IRCT2019071 9044276N1	The comparison of effect of pregabalin and duloxetine on post-op pain	No results posted
Irct201907030 44091N1	Effects of magnesium sulfate and pregabalin on postoperative pain in posterior lumbar spinal fusion surgery	Wrong intervention
ChiCTR20000 35554	The effects of pregabalin versus gabapentin combined with NSIADs on the pain of spinal surgery: a randomized controlled trial	No results posted
ChiCTR20000 31236	Effect of local infiltration using ropivacaine combined with celebrex and pregabalin on the pain control of spinal surgery	Wrong intervention
ChiCTR21000 42109	Clinical randomized controlled trial of multimodal analgesia (MMA) and patient controlled analgesia (PCA) in Oblique Lumbar Interbody Fusion	Wrong intervention

© 2023 Tsai SHL et al. *JAMA Network Open.*

eTable 3 Descriptions and Demo of the Included Studies

Study (Author, year)	Preexisting neuropathic pain	NSAID use before study	Gabapentinoid use before study	Duration of gabapentinoid use for study	Postoperative VAS assessment timing (hrs)	Postoperative opioid consumption recording timing (hrs)	Postoperative Complication assessment timing(hrs)	Comorbidities	Co-medications	Other outcomes
Pandey et al, 2004	Not mentioned	No (within 48 hrs before surgery)	Not mentioned	2 hrs before surgery	*0-6,6-12, 12-18, 18-24	*Within 24	*Within 24	NA	NA	Fatigue, light headedness
Turan et al, 2004	Not mentioned	No	No	1 hr before surgery	*1,2,4,6,12,24	1,2,4,6,12,24,*during hospitalization	*Within 24	NA	NA	Somnolence, diarrhea, pruritis, urinary retention, constipation
Pandey et al, 2005	Yes	No (within 24 hrs before surgery)	Not mentioned	2 hrs before surgery	*6,12,18,24	*Within 24	*Within 24	NA	NA	Respiratory depression, light headedness, lack of concentration, feeling on a "high"
Radhakrishnan et al, 2005	Not mentioned	No (within 24 hrs before surgery)	Not mentioned	at night before surgery and 2hrs before surgery	*2,4,6,8	*>8	*during hospitalization	NA	NA	Urinary retention, dry mouth, somnolence, pruritis, headache
Burke et al, 2010	Yes	Not mentioned	Not mentioned	1.5 hr before surgery, 12	*24	Not mentioned	*24	Smoker	Paracetamol, diclofenac, bupivacaine	McGill pain questionnaire, Roland Morris

© 2023 Tsai SHL et al. *JAMA Network Open.*

				and 24 hrs after surgery						disability questionnaire. Prolo score, Short Form 36
Hegarty et al, 2011	Yes	Yes, paracetamol 1000 mg every 6 hours	No (within 2 weeks before surgery)	1 hr before surgery	4,8,12,24	Within 24	*Within 24	NA	Diclofenac	Somnolence, lightheadedness, visual disturbance
Khan et al, 2011	Not mentioned	No (within 24 hrs before surgery)	No (within 24 hrs before surgery)	2 hrs before surgery	*0-4,4-8,8-12,12-24	*Within 24	*during hospitalization	NA	NA	Drowsiness
Kim et al, 2011	Not mentioned	No (within 48rs before surgery)	No (within 48rs before surgery)	1 hr before surgery and 12 hrs after surgery	*1-6,6-24,24-48	1-6,6-24,-24-48	*during hospitalization	NA	NA	Incidence of pain rescues, satisfaction score, sedation, headache, blurred vision
Spreng et al, 2011	Not mentioned	Yes, paracetamol 1000 mg for weight < 60kg; 1500 mg for weight >60 kg	Not mentioned	1 hr before surgery	0.5,1,2,3,4,24	0.5,1,2,3,4,24	*Within 24	NA	NA	Sedation, pruritus, urinary retention, respiratory depression, headache
Ozgencil et al, 2011	Not mentioned	No	No	2 hrs before surgery, 10 and 22 hrs after surgery	*1,2,4,6,12,24	1,2,4,6,12, *within 24	*during hospitalization	NA	NA	Satisfaction with pain medication, numeric sedation score, somnolence,

© 2023 Tsai SHL et al. *JAMA Network Open.*

										headache, blurred vision, urinary retention, pruritus, shivering
Gianesello et al, 2012	Not mentioned	Not mentioned	No	1 hr before surgery and twice a day after surgery for 2 days	1,4,8,12,24,48, 3 months, 1year	1,4,8,12,24,*48	*during hospitalization	NA	NA	Pruritus, sedation, respiratory depression, hypotension, headache, constipation, diarrhea, peripheral edema, dry mouth, blurred vision, quality of Life
Choi et al, 2013	Yes	Not mentioned	No	1hr before surgery and every 12 hrs after surgery (total 8 doses)	*12,24,48,72	Not mentioned	*0-12,12-24,24-48,48-72	NA	NA	Incidence of pain rescue, back pain, leg pain, daily activities
Kumar et al, 2013	Not mentioned	Not mentioned	No	1hr before surgery	*1,2,4,6	*6	*during hospitalization	NA	Diclofenac	Anxiety score, sedation score, drowsiness
Khurana et al, 2014	Yes	Not mentioned	No	1hr before surgery and every 8 hours	3,6,12,24,36,48, 72,7days, 21days,3 months	Not mentioned	*during hospitalization	Diabetes, hypertension, alcoholic, smoker, others	NA	Prolo score, Oswestry disability index score, sedation,

© 2023 Tsai SHL et al. *JAMA Network Open.*

				for 7 days after surgery						vertigo, numbness
Zarei et al, 2016	Yes	Yes	No(within 2 weeks before surgery)	8hrs before surgery and every 12 hours for 14 days after surgery for 2 days or14 days	*4,8,12,24	Not mentioned	Within 24	Smoker	NA	NA
Vasigh et al, 2016	Yes	No	Not mentioned	2hrs before surgery and 6 hours after surgery	*2,4,6,8,12,24	*Not mentioned	*during hospitalization	NA	NA	Shivering, drowsiness, pruritus, urinary retention, headache
Qadeer et al, 2017	Not mentioned	Not mentioned	No	twice daily for a week before surgery	*24, 1 week	Not mentioned	Not mentioned	Hypertension, diabetes, COPD, others	NA	NA
Yadav et al, 2018	Not mentioned	No (within 24hrs before surgery)	No	2hrs before surgery	Not mentioned	*during hospitalization	Not mentioned	NA	NA	Mean arterial blood pressure, heart rate, bispectral index
Urban et al, 2018	Yes	Not mentioned	No	1hr before surgery and twice daily for 14 days after surgery	*24,48,72	0-6,7-12,13-18,19-24,25-48,49-72	*24,48,72	NA	NA	Sedation, hospital stay
Altiparmak et al, 2018	Not mentioned	No	No	1hr before surgery, 12th and 24th hr after surgery	1 min, 0.5, 1,2,24,48	Not mentioned	*during hospitalization	NA	Paracetamol	Montreal Cognitive Assessment, delay of first

© 2023 Tsai SHL et al. *JAMA Network Open.*

										analgesic request, incidence of pain rescue, hypotension, bradycardia, allergia, itching
Routray et al, 2018	Not mentioned	Not mentioned	Not mentioned	1hr before surgery	1,2,4,6,8,12,16, 20,24	within 24	*during hospitalization	NA	Paracetamol	Sedation, time to first dose of rescue analgesia, incidence of pain rescues,
Raja et al, 2019	Not mentioned	No(within 24 hrs before surgery)	Not mentioned	4hrs before surgery	4,8,12,16,20,24, 28,32,36,40,44, 48	*during hospitalization	*during hospitalization	NA	Paracetamol, ketorolac	Dry mouth, difficulty in urination, constipation, hospital stay, -North American Spine Society satisfaction scale
Momon et al, 2019	Not mentioned	Not mentioned	No (within 48 hrs before surgery)	1 hr before surgery	During hospitalization, 6 months	*within 48	*During hospitalization	Hypertension, coronary disease, cardiac insufficiency, arteritis, thromboembolic disease, sleep apnea,	Acetaminophen, nonsteroidal anti-inflammatory drugs	Hospital stay, time to first ambulation, persistent pain, pain intensity, regular opioid intake, neuropathic pain, quality of

© 2023 Tsai SHL et al. *JAMA Network Open.*

								smoker, dyspnea, asthma, gastroesophageal disease, viral hepatitis, regular alcohol intake, diabetes, dyslipidemia, hypothyroidism, allergy, cancer, lithiasis, migraine, anxiety, depression, cerebral disease, infection		first attempt to stand up, sedation, urinary retention, hypotension, hypertension, sleep disturbance, headache, diarrhea, hyperglycemia, gastralgia, hyperthermia, respiratory disturbance, radiculopathy
Bala et al, 2019	Not mentioned	Not mentioned	No	1.5hr before surgery	*1,2,4,6,1,24	*within 24	During hospitalization	NA	NA	Recovery profile, Ramsay sedation scale score
Kien et al, 2019	Not mentioned	Not mentioned	No	2hrs before surgery	*0.25,0.5,1,4,8, 16,24,36,48	Within 24,*within 48	During hospitalization	NA	Celecoxib	Mean arterial blood pressure, heart rate, respiratory rate, SpO_2, sedation score, time to

© 2023 Tsai SHL et al. *JAMA Network Open.*

										first dose of rescue analgesia
Zhang et al, 2021	Not mentioned	Not mentioned	Not mentioned	2hrs before surgery twice daily for 7 days after surgery	*2,12,24,72,120	Not mentioned	*During hospitalization	NA	Celebrex, ropivacaine	Walking time, hospital stay, fever, infection, urination disorder, secondary surgery
Baloch et al, 2021	Yes	Not mentioned	Not mentioned	Twice daily on preoperative day till one week after surgery	*1 week	Not mentioned	Not mentioned	NA	Paracetamol, ketorolac	Roland-Morris disability scale

NSAIDs: Non-steroidal anti-inflammatory drugs
*: used for further analysis

© 2023 Tsai SHL et al. *JAMA Network Open.*

eTable 4: Assessment of Inconsistency With Design-by-Treatment Interaction Models

	Chi-Square	P value for test of global inconsistency
VAS	7.59	0.6684
Opioid Consumption	3.70	0.9883
Nausea	6.69	0.9461
Vomit	7.83	0.8121
Dizziness	2.67	0.9536

eTable 5 Meta-Regression of Covariates for Outcomes

Outcomes	Covariates	P value
VAS	Co-medication	0.6743
	Funding	0.7427
	Postoperative gabapentinoid use	0.3004
	Preexisting neuropathic pain	0.4208
Opioid Consumption	Co-medication	0.5127
	Funding	0.9039
	Postoperative gabapentinoid use	0.5182
	Preexisting neuropathic pain	0.0566
Nausea	Co-medication	0.9283
	Funding	0.0148*
	Postoperative gabapentinoid use	0.0513
	Preexisting neuropathic pain	0.4762
Vomit	Co-medication	0.8106
	Funding	0.2638
	Postoperative gabapentinoid use	0.3476
	Preexisting neuropathic pain	0.6426
Dizziness	Co-medication	0.7637
	Funding	0.9878
	Postoperative gabapentinoid use	0.8308
	Preexisting neuropathic pain	0.7637
*significant difference		

© 2023 Tsai SHL et al. *JAMA Network Open.*

eTable 6 SUCRA, PrBest, and Mean Rank of Different Dosages of Gabapentin and Pregabalin for Patients Undergoing Spinal Surgery.

A

Treatment(**Vomit**)	SUCRA	PrBest	MeanRank
Gabapentin900mg	70.8	35.4	3.3
Pregabalin150mg	69.9	22.2	3.4
Gabapentin1200mg	63.0	10.4	4.0
Gabapentin600mg	62.4	6.5	4.0
Pregabalin300mg	49.2	2.1	5.1
Pregabalin75mg	48.7	5.7	5.1
Gabapentin800mg	45.3	16.4	5.4
Gabapentin300mg	27.1	1.3	6.8
Placebo	13.5	0.0	7.9

B

Treatment(**Dizziness**)	SUCRA	PrBest	MeanRank
Gabapentin300mg	77.9	60.6	2.5
Pregabalin150mg	64.7	17.5	3.5
Placebo	63.2	2.7	3.6
Pregabalin300mg	53.8	2.8	4.2
Gabapentin900mg	49.1	12.8	4.6
Gabapentin600mg	34.0	1.3	5.6
Gabapentin1200mg	30.2	0.6	5.9
Pregabalin75mg	27.3	1.7	6.1

© 2023 Tsai SHL et al. *JAMA Network Open.*

Sensitivity analysis for nausea (studies without funding)

eFigure 10: Network Graph for Nausea Without Funding

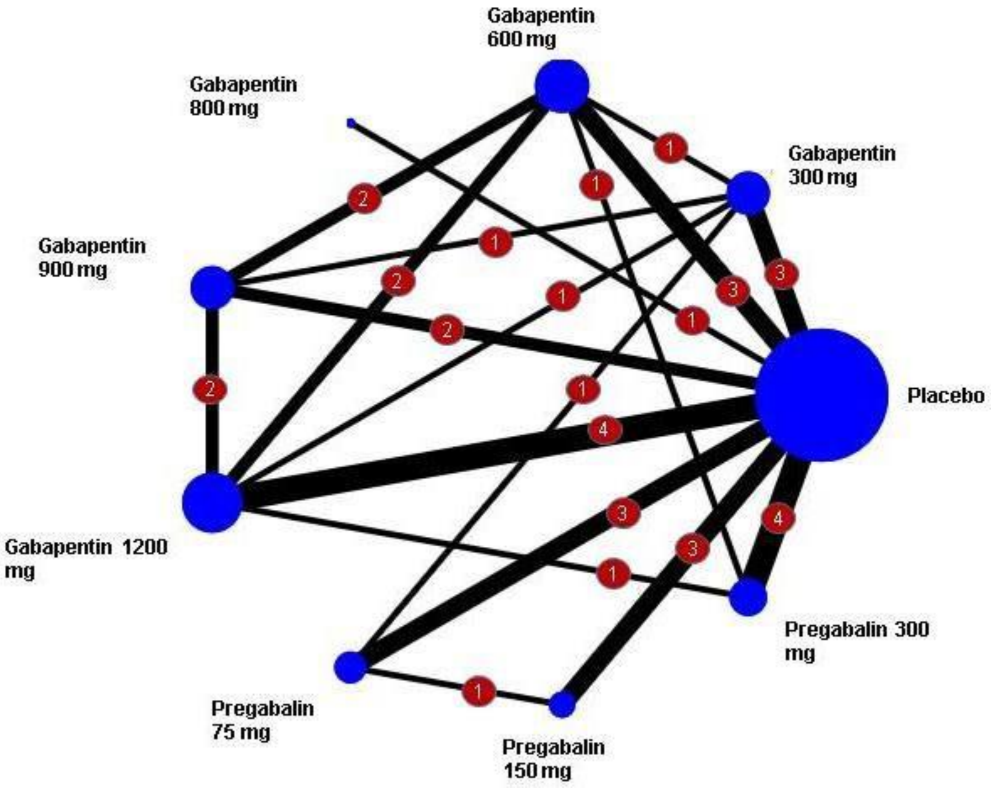

© 2023 Tsai SHL et al. *JAMA Network Open.*

eFigure 11: Network Meta-Analysis League Tables for Nausea Without Funding

(In the left lower half, odds ratio lower than 1 favor the row-defining treatment)

Pregabalin75								
3.00 (0.97,9.31)	Pregabalin300							
3.10 (0.90,10.64)	1.03 (0.32,3.36)	Pregabalin150						
1.72 (0.71,4.20)	0.57 (0.28,1.16)	0.56 (0.21,1.44)	Placebo					
2.05 (0.42,10.02)	0.68 (0.16,2.90)	0.66 (0.13,3.37)	1.19 (0.32,4.47)	Gabapentin900				
1.72 (0.37,8.10)	0.57 (0.14,2.44)	0.56 (0.11,2.71)	1.00 (0.28,3.54)	0.84 (0.13,5.25)	Gabapentin800			
1.58 (0.42,5.97)	0.53 (0.17,1.61)	0.51 (0.13,2.02)	0.92 (0.34,2.48)	0.77 (0.19,3.07)	0.92 (0.18,4.59)	Gabapentin600		
2.41 (0.72,8.09)	0.80 (0.23,2.83)	0.78 (0.19,3.17)	1.40 (0.48,4.03)	1.18 (0.22,6.15)	1.40 (0.27,7.28)	1.53 (0.37,6.33)	Gabapentin300	
1.63 (0.52,5.07)	0.54 (0.22,1.31)	0.52 (0.16,1.72)	0.94 (0.46,1.93)	0.79 (0.21,3.07)	0.94 (0.22,4.04)	1.03 (0.35,3.05)	0.68 (0.19,2.36)	Gabapentin1200

© 2023 Tsai SHL et al. *JAMA Network Open.*

eFigure 12: Rank Plot for Nausea Without Funding

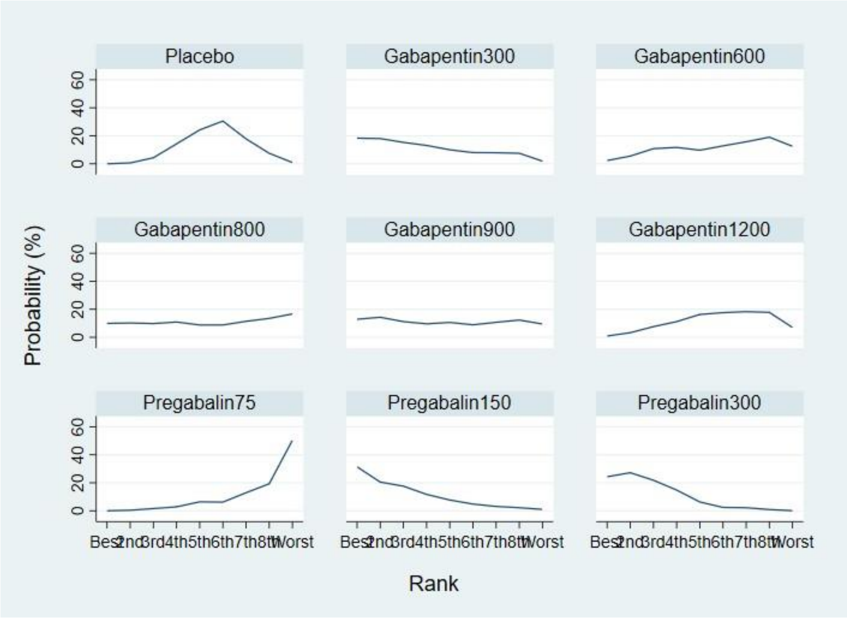

eFigure 13: Forest Plot of Network Meta-Analysis Results for Nausea Without Funding (Placebo as Reference)

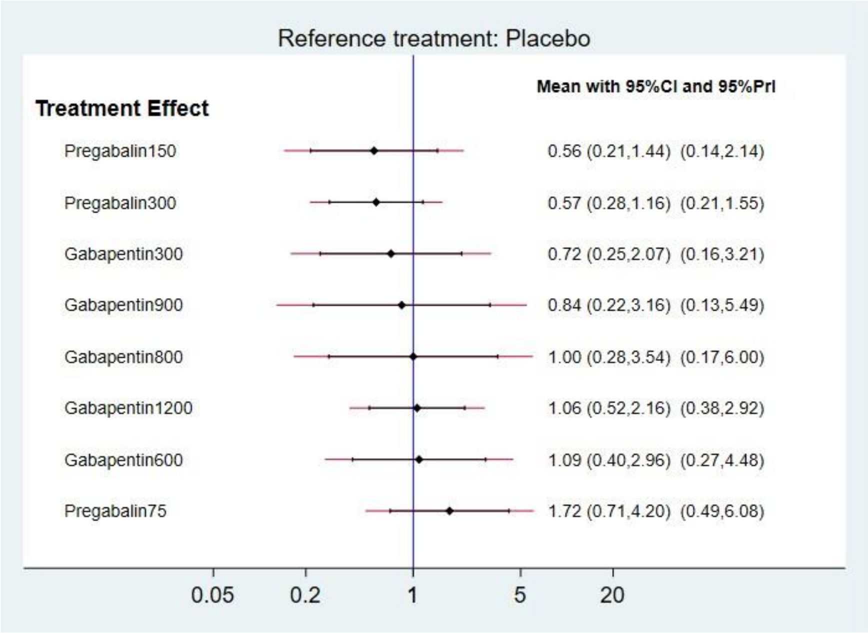

© 2023 Tsai SHL et al. *JAMA Network Open.*

eFigure 14: Forest Plot of Pairwise Comparison for Nausea Without Funding

(A: placebo, B: Gabapentin 300mg, C: Gabapentin 600 mg, D: Gabapentin 800mg, E: Gabapentin 900mg, F: Gabapentin 1200mg, G: pregabalin 75mg, H: pregabalin 150mg, I: pregabalin 300mg)

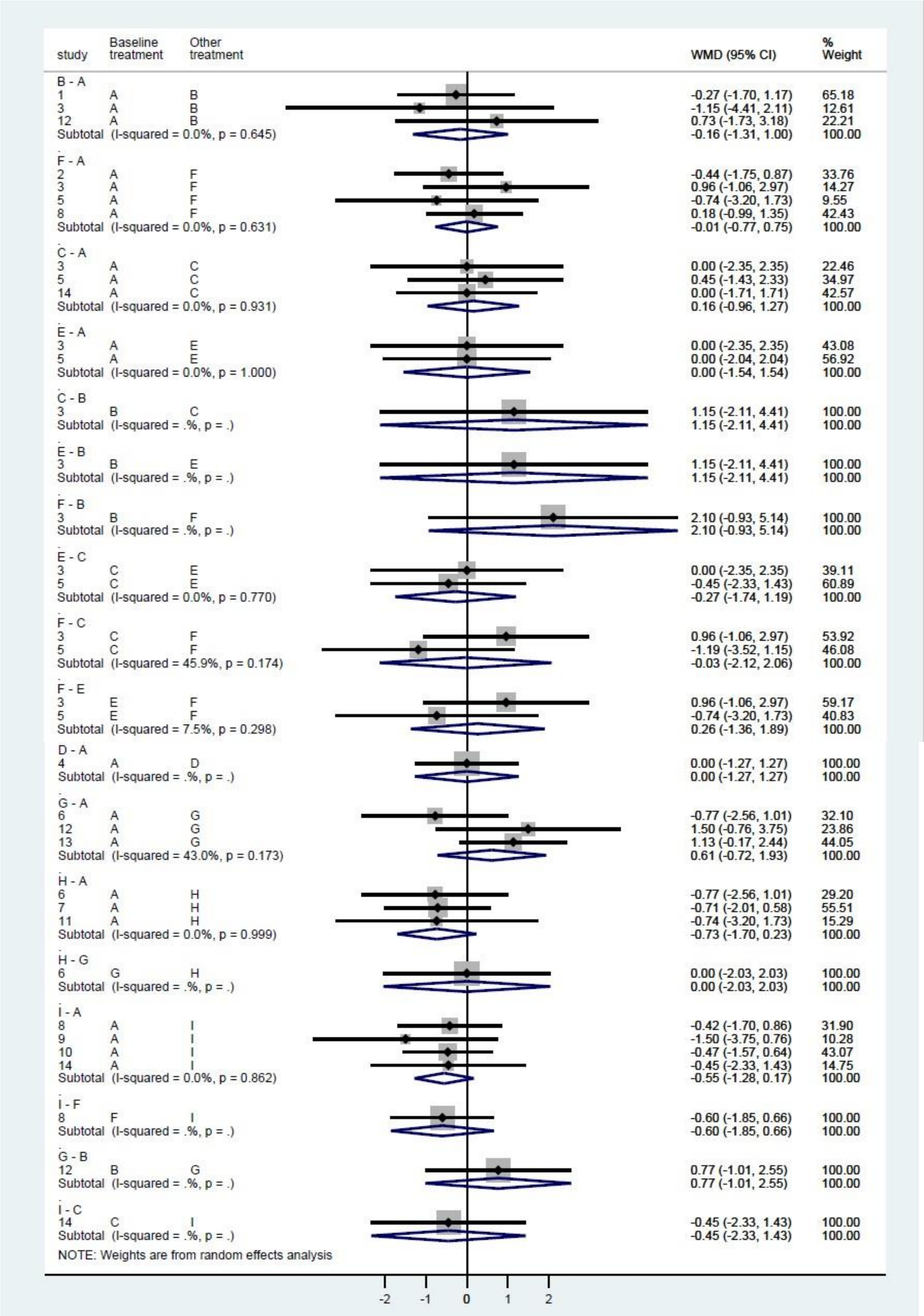

© 2023 Tsai SHL et al. *JAMA Network Open.*

eFigure 15: Publication Bias: Funnel Plot for Nausea Without Funding

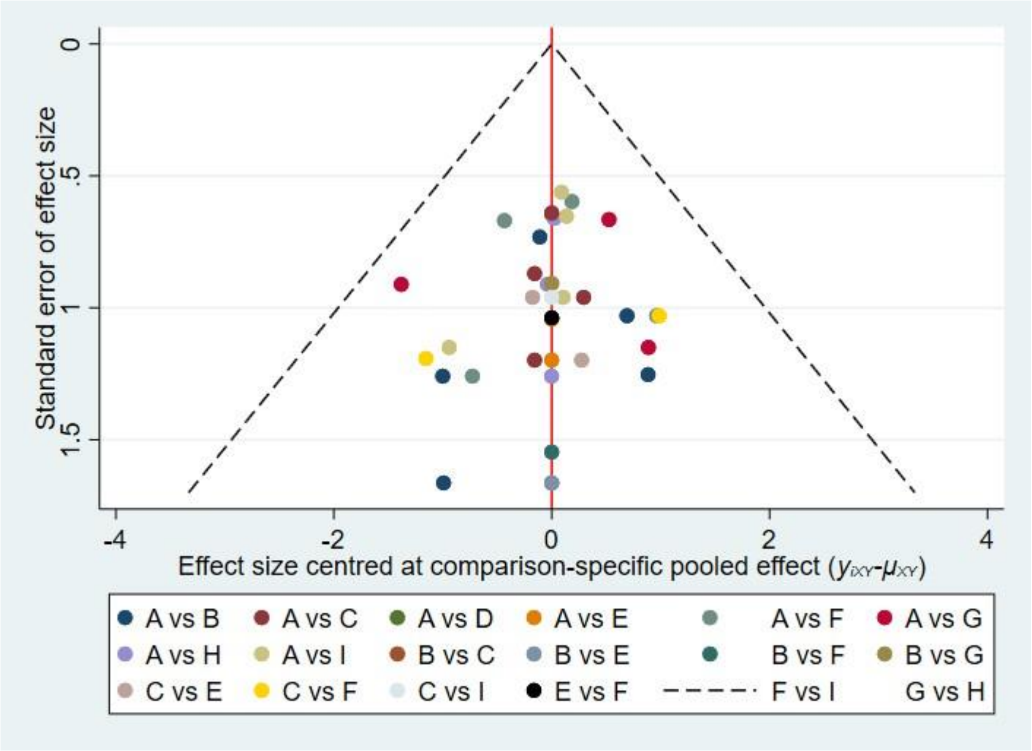

eFigure 16: Publication Bias: The Egger Test for Nausea Without Funding

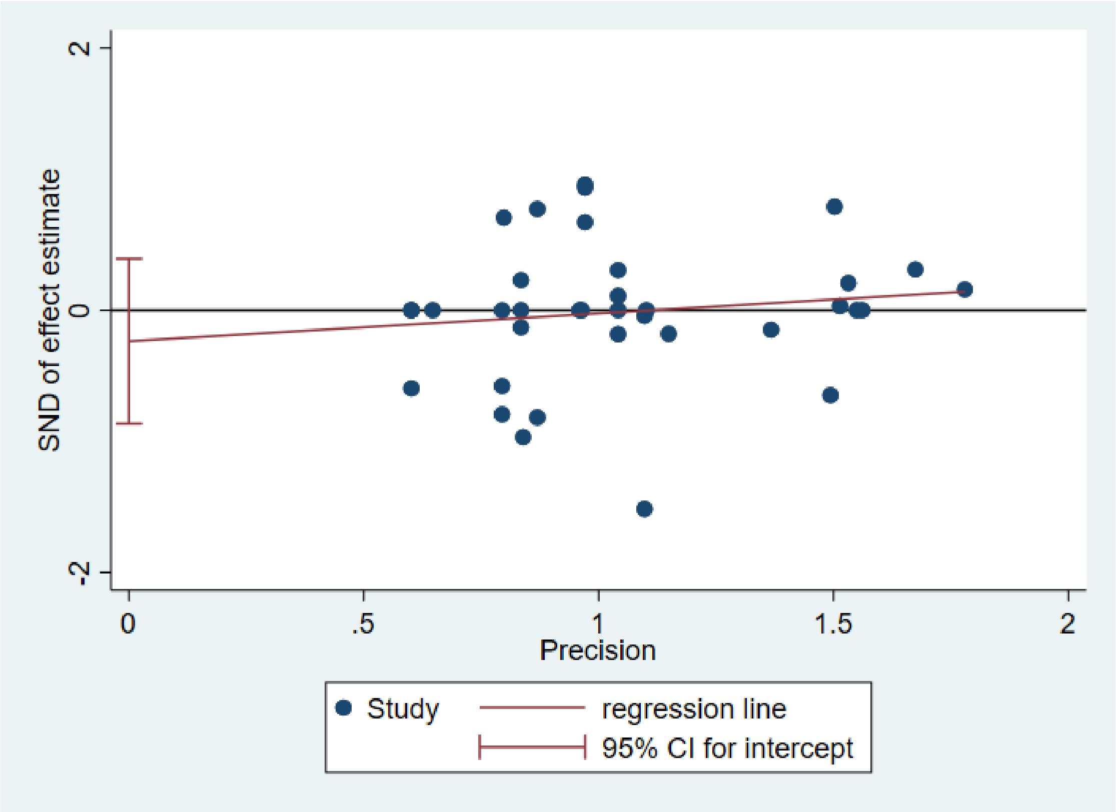

© 2023 Tsai SHL et al. *JAMA Network Open*.

eFigure 17: CINeMA for Nausea Without Funding

Comparison	Number of Studies	Within-study bias	Reporting bias	Indirectness	Imprecision	Heterogeneity	Incoherence	Confidence rating
Mixed evidence								
Gabapentin1200 vs Gabapentin300	1	Some concerns	Low risk	No concerns	Major concerns	No concerns	No concerns	Very low
Gabapentin1200 vs Gabapentin600	2	Some concerns	Low risk	No concerns	Major concerns	No concerns	No concerns	Very low
Gabapentin1200 vs Gabapentin900	2	Some concerns	Low risk	No concerns	Major concerns	No concerns	No concerns	Very low
Gabapentin1200 vs Placebo	4	Some concerns	Low risk	No concerns	Major concerns	No concerns	No concerns	Very low
Gabapentin1200 vs Pregabalin300	1	Some concerns	Low risk	No concerns	Major concerns	No concerns	No concerns	Very low
Gabapentin300 vs Gabapentin600	1	Some concerns	Low risk	No concerns	Major concerns	No concerns	No concerns	Very low
Gabapentin300 vs Gabapentin900	1	Some concerns	Low risk	No concerns	Major concerns	No concerns	No concerns	Very low
Gabapentin300 vs Placebo	3	No concerns	Low risk	No concerns	Major concerns	No concerns	No concerns	Low
Gabapentin300 vs Pregabalin75	1	Some concerns	Low risk	No concerns	Major concerns	No concerns	No concerns	Very low
Gabapentin600 vs Gabapentin900	2	Some concerns	Low risk	No concerns	Major concerns	No concerns	No concerns	Very low
Gabapentin600 vs Placebo	3	Some concerns	Low risk	No concerns	Major concerns	No concerns	No concerns	Very low
Gabapentin600 vs Pregabalin300	1	Some concerns	Low risk	No concerns	Major concerns	No concerns	No concerns	Very low
Gabapentin800 vs Placebo	1	No concerns	Low risk	No concerns	Major concerns	No concerns	No concerns	Low
Gabapentin900 vs Placebo	2	Some concerns	Low risk	No concerns	Major concerns	No concerns	No concerns	Very low
Placebo vs Pregabalin150	3	Some concerns	Low risk	No concerns	Major concerns	No concerns	No concerns	Very low
Placebo vs Pregabalin300	4	No concerns	Low risk	No concerns	Major concerns	No concerns	No concerns	Low
Placebo vs Pregabalin75	3	No concerns	Low risk	No concerns	Major concerns	No concerns	No concerns	Low
Pregabalin150 vs Pregabalin75	1	No concerns	Low risk	No concerns	Major concerns	No concerns	No concerns	Low
Indirect evidence								
Gabapentin1200 vs Gabapentin800	--	No concerns	Low risk	No concerns	Major concerns	No concerns	No concerns	Low
Gabapentin1200 vs Pregabalin150	--	Some concerns	Low risk	No concerns	Major concerns	No concerns	No concerns	Very low
Gabapentin1200 vs Pregabalin75	--	No concerns	Low risk	No concerns	Major concerns	No concerns	No concerns	Low
Gabapentin300 vs Gabapentin800	--	No concerns	Low risk	No concerns	Major concerns	No concerns	No concerns	Low
Gabapentin300 vs Pregabalin150	--	No concerns	Low risk	No concerns	Major concerns	No concerns	No concerns	Low
Gabapentin300 vs Pregabalin300	--	No concerns	Low risk	No concerns	Major concerns	No concerns	No concerns	Low
Gabapentin600 vs Gabapentin800	--	Some concerns	Low risk	No concerns	Major concerns	No concerns	No concerns	Very low
Gabapentin600 vs Pregabalin150	--	Some concerns	Low risk	No concerns	Major concerns	No concerns	No concerns	Very low
Gabapentin600 vs Pregabalin75	--	Some concerns	Low risk	No concerns	Major concerns	No concerns	No concerns	Very low
Gabapentin800 vs Gabapentin900	--	Some concerns	Low risk	No concerns	Major concerns	No concerns	No concerns	Very low
Gabapentin800 vs Pregabalin150	--	No concerns	Low risk	No concerns	Major concerns	No concerns	No concerns	Low
Gabapentin800 vs Pregabalin300	--	No concerns	Low risk	No concerns	Major concerns	No concerns	No concerns	Low
Gabapentin800 vs Pregabalin75	--	No concerns	Low risk	No concerns	Major concerns	No concerns	No concerns	Low
Gabapentin900 vs Pregabalin150	--	Some concerns	Low risk	No concerns	Major concerns	No concerns	No concerns	Very low
Gabapentin900 vs Pregabalin300	--	Some concerns	Low risk	No concerns	Major concerns	No concerns	No concerns	Very low
Gabapentin900 vs Pregabalin75	--	Some concerns	Low risk	No concerns	Major concerns	No concerns	No concerns	Very low
Pregabalin150 vs Pregabalin300	--	Some concerns	Low risk	No concerns	Major concerns	No concerns	No concerns	Very low
Pregabalin300 vs Pregabalin75	--	No concerns	Low risk	No concerns	Major concerns	No concerns	No concerns	Low

© 2023 Tsai SHL et al. *JAMA Network Open.*

eTable 7. SUCRA, PrBest, and Mean Rank of Different Dosages of Gabapentin and Pregabalin for Patients Undergoing Spinal Surgery.

Treatment (**Nausea without funding**)	SUCRA	PrBest	MeanRank
Pregabalin300mg	78.4	24.3	2.7
Pregabalin150mg	76.6	31.4	2.9
Gabapentin300mg	64.6	18.3	3.8
Gabapentin900mg	52.7	12.9	4.8
Gabapentin800mg	45.2	9.9	5.4
Placebo	41.6	0.0	5.7
Gabapentin600mg	38.5	2.3	5.9
Gabapentin1200mg	38.0	0.9	6.0
Pregabalin75mg	14.5	0.0	7.8

eTable 8: Assessment of Inconsistency With Design-by-Treatment Interaction Models

	Chi-Square	P value for test of global inconsistency
VAS	7.53	0.8728

eReferences

1. Myles, P. S. *et al.* Measuring acute postoperative pain using the visual analog scale: the minimal clinically important difference and patient acceptable symptom state. *Br. J. Anaesth.* **118**, 424–429 (2017).

2. Papakonstantinou, T., Nikolakopoulou, A., Higgins, J. P. T., Egger, M. & Salanti, G. CINeMA: Software for semiautomated assessment of the confidence in the results of network meta-analysis. *Campbell Syst. Rev.* **16**, (2020).

© 2023 Tsai SHL et al. *JAMA Network Open.*
