# Supplementary material for: Different Gabapentin and Pregabalin Dosages for Perioperative Pain Control in Patients Undergoing Spine Surgery: A Systematic Review and Network Meta-Analysis
Source: JAMA Netw Open. 2023 Aug 9;6(8):e2328121. doi: 10.1001/jamanetworkopen.2023.28121 (PMC10413173; doi:10.1001/jamanetworkopen.2023.28121)
Supplement: Supplement 2. — Data Sharing Statement [file jamanetwopen-e2328121-s002.pdf]

Data Sharing Statement

Tsai. Different Gabapentin and Pregabalin Dosages for Perioperative Pain Control in Patients Undergoing Spine Surgery. *JAMA Netw Open*. Published August 09, 2023. doi:10.1001/jamanetworkopen.2023.28121

Data
Data available: No
